# Reconfigurable asymmetric protein assemblies through implicit negative design

**DOI:** 10.1126/science.abj7662

**Published:** 2022-01-21

**Authors:** Danny D. Sahtoe, Florian Praetorius, Alexis Courbet, Yang Hsia, Basile I.M. Wicky, Natasha I. Edman, Lauren M. Miller, Bart J. R. Timmermans, Justin Decarreau, Hana M. Morris, Alex Kang, Asim K. Bera, David Baker

**Affiliations:** 1 Department of Biochemistry, University of Washington, Seattle, WA 98195; 2 Institute for Protein Design, University of Washington, Seattle, WA 98195; 3 HHMI, University of Washington, Seattle, WA 98195; 4 Molecular and Cellular Biology Graduate Program, University of Washington, Seattle, WA, USA.; 5 Medical Scientist Training Program, University of Washington, Seattle, WA, USA

## Abstract

Asymmetric multi-protein complexes that undergo subunit exchange play central roles in biology, but present a challenge for design since the components must contain interfaces enabling reversible association but be stable and well behaved in isolation. We use implicit negative design to generate beta sheet mediated heterodimers which can be assembled into a wide variety of complexes. The designs are stable, folded and soluble in isolation and rapidly assemble upon mixing, and crystal structures are close to the computational models. We construct linearly arranged hetero-oligomers with up to 6 unique components, branched hetero-oligomers, closed C4-symmetric two-component rings, and hetero-oligomers assembled on a cyclic homo-oligomeric central hub, and demonstrate such complexes can readily reconfigure through subunit exchange. Our approach provides a general route to designing asymmetric reconfigurable protein systems.

## One-Sentence Summary

De novo designed protein building blocks can be modularly combined to create customized protein assemblies.

Dynamic reconfigurable multi-protein complexes play key roles in central biological processes (1). The subunits are generally monomeric in isolation, allowing the assemblies to reconfigure by successive addition or removal of one or more components. Such modulation is essential to their function: for example, subunit loss and addition underlies the molecular mechanisms of protein complexes that drive DNA replication and transcription (2, 3). The ability to de novo design such multicomponent reconfigurable protein assemblies would enable the realization of sophisticated new functions. Previous design efforts have generated cyclic oligomeric and higher order symmetric nanostructures such as icosahedral nanocages with as many as 120 subunits, and 2D-layers with many thousands of regularly arrayed components (4–8). Essential to this is the symmetry and cooperativity of assembly, which strongly favors just one of a large number of possible states. Once formed, these assemblies are therefore typically quite static and exchange subunits only on long time scales, which is advantageous for applications such as nanoparticle vaccine design and multivalent receptor engagement (9).

The design of reconfigurable asymmetric assemblies is more challenging, as there is no symmetry “bonus” favoring the target structure (as is attained for example in the closing of an icosahedral cage), and because the individual subunits must be stable and soluble proteins in isolation in order to reversibly associate. Reconfigurable asymmetric protein assemblies could in principle be constructed using a modular set of protein-protein interaction pairs (heterodimers), provided first, that the individual subunits are stable and monomeric in isolation so they can be added and removed, second, that the interacting pairs are specific, and third, that they can be rigidly fused through structured connectors to other components. Rigid fusion, as opposed to fusion by flexible linkers, is important to program the assembly of structurally well defined complexes; most higher order natural protein complexes have, despite their reconfigurability, distinct overall shapes critical for their function. While there are designed orthogonal sets of interacting proteins that have one of these properties, designed proteins having all of these properties are lacking. The components of designed helical-hairpin heterodimers (10, 11) on their own form homodimers or other higher order homomeric aggregates that disassemble on very long time scales (10, 12), making them unsuitable for use in constructing reconfigurable higher order assemblies. Heterodimeric coiled-coils assemble from peptides that are soluble and monomeric, but the monomers are unfolded prior to binding their partners (13, 14), complicating their use in structurally defined rigid fusions.

**Fig. 1.**
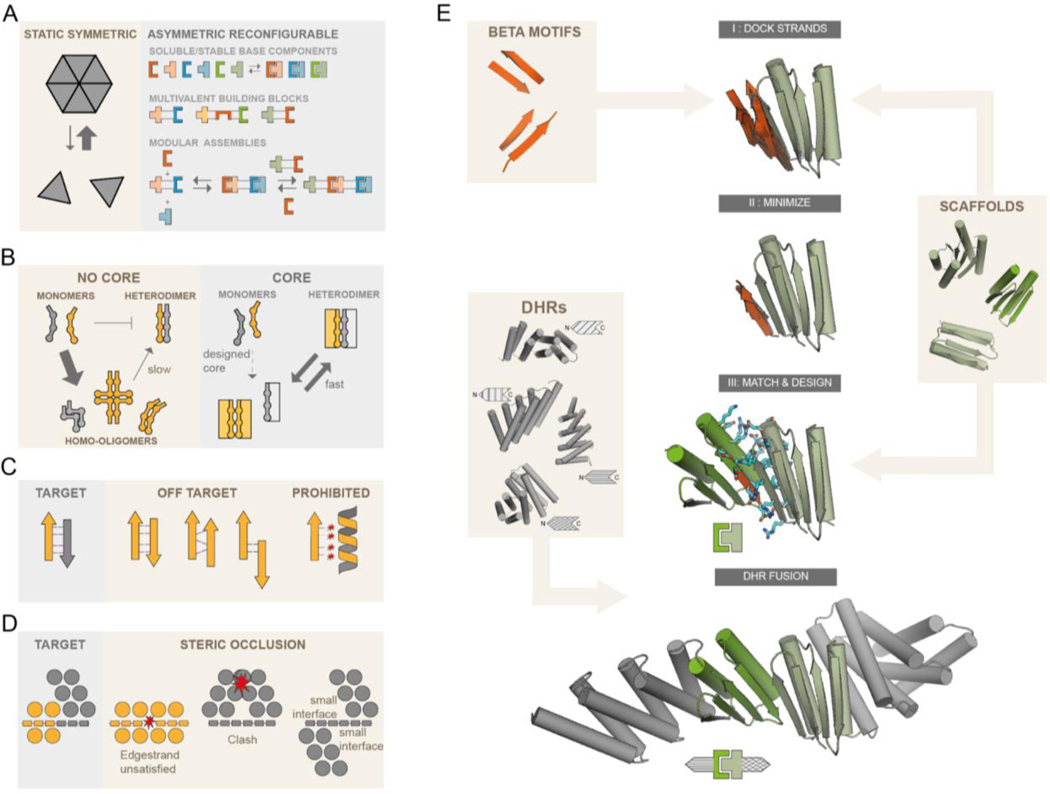
Strategies for the design of asymmetric hetero-oligomeric complexes. **(A)** Many design efforts have focused on cooperatively assembling symmetric complexes (left) with little subunit exchange. Here we sought to create asymmetric hetero-oligomers from stable heterodimeric building blocks, that can modularly exchange subunits (right). **(B,C,D)** Schematic illustration of properties that can contribute to preventing self-association. **(B)** Protomers that have a substantial hydrophobic core (right rectangles) are less likely to form stable homo-oligomers than protomers of previously designed heterodimers lacking hydrophobic monomer cores. **(C)** In beta-sheet extended interfaces, most homodimer states that bury non h-bonding polar edge strand atoms are energetically inaccessible. Potential homodimers are more likely to form via beta sheet extension. These are restricted to only 2 orientations (parallel and antiparallel) and a limited number of offset registers. Arrows and ribbons represent strands and helices, respectively; thin lines indicate hydrogen bonds, red stars indicate unsatisfied polar groups. **(D)** “Cross sectional” schematic view (helices as circles, beta strands as rectangles, star indicates steric clash) By modeling the limited number of beta sheet homodimers across the beta edge strand, structural elements may be designed that specifically block homodimer formation or make it unlikely due to small interfaces, but still allow heterodimer formation. **(E)** Design workflow: Beta sheet motifs are docked to the edge strands of a library of hydrophobic core containing (modified) fold-it scaffolds. Minimized docked strands are incorporated into the scaffolds by matching the strands to the scaffold library, yielding docked protein-protein complexes, followed by interface sequence design. Resulting docks are fused rigidly on their terminal helices to a library of DHRs.

We set out to design sets of interacting protein pairs for constructing reconfigurable assemblies (Fig. 1A). The first challenge is the systematic design of proteins with interaction surfaces that drive association with cognate partners, but not self association. Hydrophobic interactions drive protein complex assembly, but these same hydrophobic interactions can also promote homomerization. Previously designed heterodimeric helical bundles featured, in addition to hydrophobic interactions, explicit hydrogen bond networks that contribute to binding specificity and make the interface more polar. However, the individual protomers, either helical hairpins or individual helices, lack a hydrophobic core and are thus flexible and unstable as monomers, allowing a wide range of potential off-target homo-oligomers to form (Fig. 1B). Explicit negative design methods favor one state by considering the effect of amino acid substitutions on the free energies of both states (15–17). However, such methods cannot be readily applied to disfavor self association, as there are in general a large number of possible self associated states which cannot be systematically enumerated.

We instead sought to use implicit negative design (18) by introducing three properties that collectively make self associated states unlikely to have low free energy: First, in contrast to the flexible coiled coils and helical hairpins in previous designs, we aimed for well folded individual protomers stabilized by substantial hydrophobic cores; this property limits the formation of slowly-exchanging homo-oligomers (Fig. 1B). Second, we constructed interfaces in which each protomer has a mixed alpha-beta topology and contributes one exposed beta strand to the interface, giving rise to a continuous beta sheet across the heterodimer interface (19–21) (Fig. 1C). The exposed polar backbone atoms of this “edge strand” limit self-association to arrangements that pair the beta edge strands; most other homomeric arrangements are unlikely because they result in energetically unfavorable burial of the polar backbone atoms on the beta edge strand (Fig. 1C). Third, taking advantage of the restrictions in possible undesired states resulting from properties 1 and 2, we explicitly modeled the limited number of homo-oligomeric states, and designed in additional elements likely to sterically occlude such states (Fig. 1D).

To implement these properties, we chose to start with a set of mixed alpha/beta scaffolds that were designed by FoldIt players (22). The selected designs contain sizable hydrophobic cores, exposed edge strands required for beta sheet extension (19) and one terminal helix (either N or C) available for rigid helical fusion (Fig. 1E) (23). Using blueprint-based backbone building (24, 25) we designed additional helices at the other terminus for a subset of the scaffolds to enable rigid fusion at both the N and C termini (Fig. S1). Heterodimers with paired beta strands across the interface were generated by superimposing one of the two strands from each of a series of paired beta strand templates onto an edge beta strand of each scaffold (Fig. 1E, top), and then optimizing the rigid body orientation and the internal geometry of the partner beta strand of the template to maximize hydrogen bonding interactions across the interface (Fig. 1E, second row). This generates a series of disembodied beta strands forming an extended beta sheet for each scaffold; for each of these, an edge beta strand from a second scaffold was superimposed on the disembodied beta strand to form an extended beta sheet (Fig. 1E, third row). The interface sidechain-sidechain interactions in the resulting protein-protein docks were optimized using Rosetta combinatorial sequence design (26). To limit excessive hydrophobic interactions, we generated explicit hydrogen bond networks across the heterodimer interface (11) or constrained the amino acid composition to favor polar residues while penalizing buried unsatisfied polar groups (27). This resulted in interfaces that, outside of the polar hydrogen bonding of the beta strands, contained both hydrophobic interactions and polar networks. To further disfavor unwanted homodimeric interactions (Fig. 1D, right panel) we rigidly fused designed helical repeat proteins (DHRs) to terminal helices (23, 28). Since these DHRs have different shapes, they also serve to diversify building block shapes for subsequent higher order assembly design. Designed heterodimers were selected for experimental characterization based on binding energy, the number of buried unsatisfied polar groups, buried surface area and shape complementarity (see methods).

**Fig 2.**
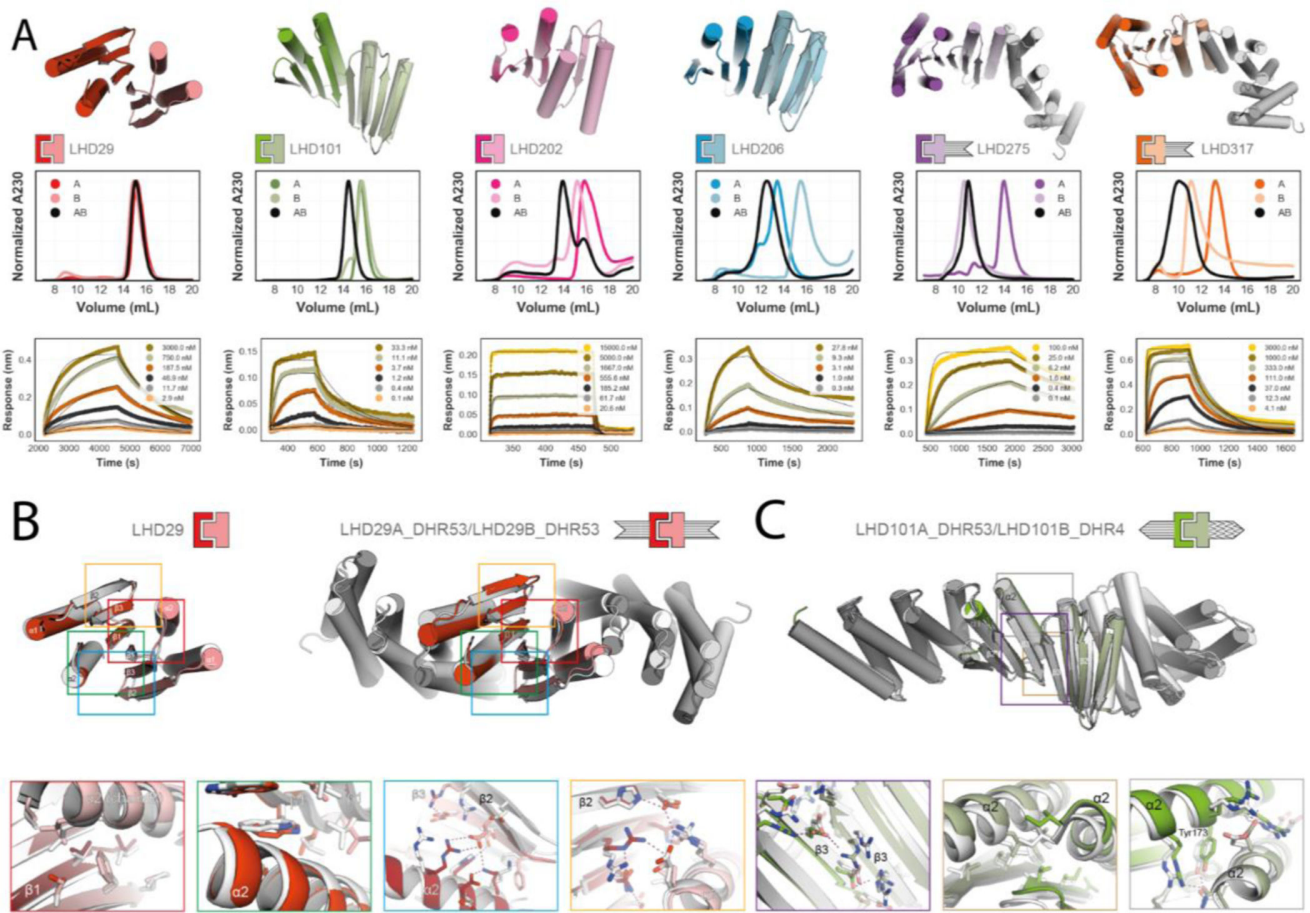
Designed heterodimer characterization. **(A)** Top row, design models of six different heterodimers. Coloring of heterodimer schematics is maintained throughout the paper. Middle row, normalized SEC traces of individual protomers (A, B) and complexes (AB). Bottom row, kinetic binding traces with global kinetic fits of in vitro biolayer interferometry binding assays. **(B) and (C):** Crystal structures (in colors) of the designs LHD29, LHD29A53/B53 and LHD101A53/B4 overlayed on design models (light gray). Colored rectangles in the full models (top row) match the corresponding detailed views (bottom row). Sequences and models for all proteins can be found in the Supplementary excel file.

We co-expressed the selected heterodimers in *E. coli* using a bicistronic expression system encoding one of the two protomers with a C-terminal polyhistidine tag and the other either untagged or GFP-tagged at the N-terminus. Complex formation was initially assessed using nickel affinity chromatography; designs for which both protomers were present in SDS-PAGE after nickel pulldown were subjected to size exclusion chromatography (SEC) and liquid chromatography - mass spectrometry (LC/MS). Of the 238 tested designs, 71 passed the bicistronic screen and were selected for individual expression of protomers. Of these, 32 formed heterodimers from individually purified monomers as confirmed by SEC, native MS, or both (Fig. 2A, Fig. S2 and S3A). In SEC titration experiments, some protomers were monomeric at all injection concentrations, while others self-associated at higher concentrations (Fig. S4). Both LHD101 protomers and their fusions were monomeric even at injection concentrations above 100 μM (Fig. S4). LHD275A, LHD278A, LHD317A, and a redesigned version of LHD29 with a more polar interface (LHD274) were also predominantly monomeric (Fig. S4; Fig. S5). Designs for which isolated protomers were poorly expressed, polydisperse in SEC or did not yield stable, soluble and functional rigid DHR fusions were discarded together with designs that were very similar to other designs, but otherwise stable and soluble. The remaining 11 heterodimers span three main structural classes (Fig. 2A, Fig. S2, supplementary excel file 1 (LHD components)). In class one, the central extended beta sheet is buttressed on opposite sides by helices that contribute additional interface interactions (LHDs 29 and 202 in Fig. 2A), in class two the helices that provide additional interactions are on the same side of the extended central sheet (LHDs 101 and 206 in Fig. 2A), and in the third class, both sides of the central beta sheet extension are flanked by helices (LHDs 275 and 317 in Fig. 2A).

We monitored the kinetics of heterodimer formation and dissociation through biolayer interferometry (BLI) (Fig. 2A, Fig. S2, and table S1) by immobilizing individual biotinylated protomers onto streptavidin coated sensors and adding the designed binding partner. Unlike previously designed heterodimers, binding reactions equilibrated rapidly, with affinities ranging from micromolar to low nanomolar (Fig. S3C and Table S1). Association rates were quite fast and ranged from 10^6^ M^−1^ s^−1^ for the fastest heterodimer to 10^2^ M^−1^ s^−1^ for the slowest heterodimer LHD29, which is still an order of magnitude faster than the fastest associating designed helical hairpin heterodimer DHD37 (10) (Fig. 2A, Fig. S6A, Table S1–2). For LHD101 and LHD206 we independently determined *K*_d_ with a split luciferase-based binding assay in E.coli lysates, and obtained very similar values, indicating that heterodimer association is not affected by high concentrations of non-cognate proteins (Fig. S6D,E and Table S3).

We determined the crystal structures of two class one designs, LHD29 (2.2 Å) and LHD29A53/B53 (2.6 Å) in which both protomers are fused to DHR53 (Fig. 2B and Table S4). In the central extended beta sheet, the LHD29 design closely matches the crystal structure (Fig. 2B, red and green box and Table S5). Aside from backbone beta sheet hydrogens bonds, this part of the interface is supported by primarily hydrophobic packing interactions between the side chains of each interface beta edge strand. The two flanking helices on opposite sides of the central beta sheet (Fig. 2B, blue and orange box) contribute predominantly polar contacts to the interface, and are also similar in the crystal structure and design model. Apart from crystal contact induced subtle backbone rearrangements in strand 2 of LHD29B that promote the formation of a polar interaction network (Fig. 2B, blue box), most interface sidechain-sidechain interactions agree with the design model. As for unfused LHD29, the interface of LHD29A53/B53 resembles the designed model; at the fusion junction and repeat protein regions, deviations are slightly larger (Table S5).

We also determined the structure of a class two design, LHD101A53/B4 (2.2 Å), in which protomer A is fused to DHR53 and B to DHR4 (Fig. 2C and Tables S4 and S5). The crystal structure agrees well with the design model at both the interface and fusion junction, as well as the repeat protein region. In class two designs, the interface beta strand pair is reinforced by flanking helices that, unlike class one designs, are in direct contact with both each other and the interface beta sheet. The solvent exposed side of the beta interface consists primarily of electrostatic interactions (Fig. 2C, purple box) while the buried side consists exclusively of hydrophobic side chains. Together with apolar side chains on the flanking helices of both protomers, these residues form a closely packed core interface (Fig. 2C, brown box) that is further stabilized by solvent exposed polar interactions between the flanking helices. Notably, the designed semi-buried polar interaction network centered on Tyr173 is recapitulated in the crystal structure (Fig. 2C, gray box).

As described above, the third of our implicit negative design principles was to incorporate structural elements incompatible with beta sheet extension in homo-dimeric species (Fig. 1D). To assess the utility of this principle, we took advantage of the limited number of possible off target edge strand interactions that can form (Fig. 1C), and docked all protomers against themselves on the edge strand that participates in the heterodimer interface and calculated the Rosetta binding energy after relaxing of the resulting homodimeric dock (Fig. S7). Homodimer docks of the protomers that chromatographed as monomers in SEC had unfavorable energies compared to those that showed evidence of self association in agreement with our initial hypothesis (Fig. 1D), and visual inspection of these docks suggested that homodimerization was likely prevented by the presence of sterically blocking secondary structure elements (Fig. S7).

**Fig. 3.**
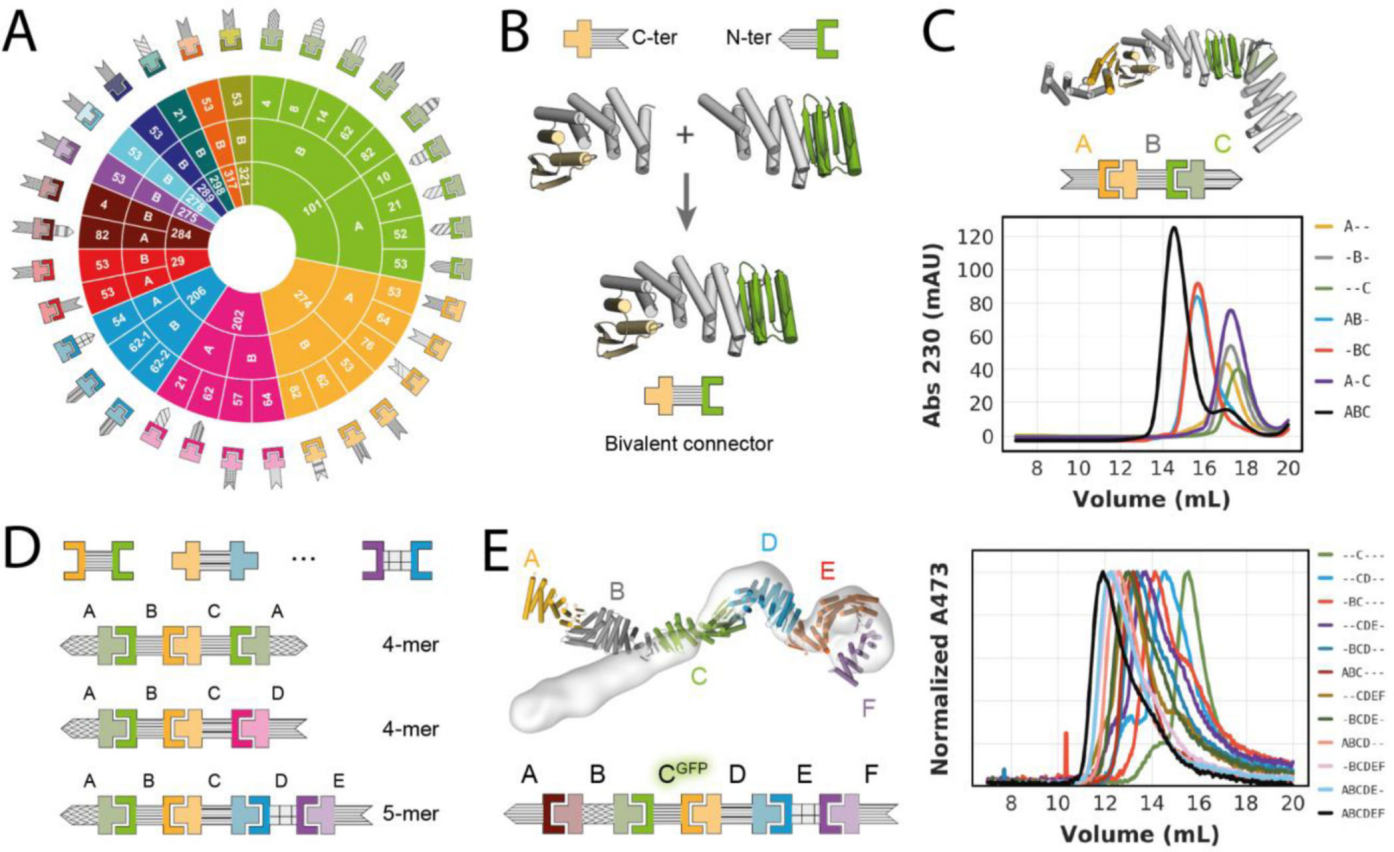
Design of higher order assemblies. **(A)** Schematic overview of experimentally validated heterodimer-DHR fusions. Inner ring represents the heterodimer, middle ring the protomer chain that is fused, and outer ring the DHR (28) fusion partner. Patterning of DHR schematic is consistent throughout the paper. **(B)** Schematic representation of the design-free alignment method used to generate bivalent connectors from rigid fusions shown in A. Top left: LHD274B fused to the N-terminus of DHR53 (274B53), Top right: LHD101A fused to the C-terminus of DHR53 (101A53), bottom: Bivalent connector DFB0. **(C)** Top: Design model and schematic representation of a heterotrimer comprising the bivalent connector shown in B (“B”), and two of the rigid fusions shown in A (“A” = 274A53; “C” = 101B62). Bottom: SEC traces for all possible combinations of the trimer components. **(D)** Schematic representations of 3 examples of bivalent connectors (see Fig. S10A for full list) that were generated as shown in B and schematic representation of experimentally validated higher order assemblies (see Fig. S10 and S11). **(E)** Left: overlay of heterohexamer design model (in colors) and nsEM density (light grey). Right: SEC traces of partial and full mixtures of the hexamer components (“A” = 284A82, “B” = DF284, “C” = DFA-GFP, “D” = DF206, “E” = DF275A, “F”=275B). Absorbance was monitored at 473 nm to follow the GFP-tagged component C. Sequences, models and chain-to-construct mapping can be found in the Supplementary excel file. Affinities of individual interactions can be found in Supplementary tables S1 and S3. Mapping of schemes to names for individual components can be found in Fig. S25.

28 additional rigid fusion proteins generated using the 11 base heterodimers and LHD274 (Fig. 3A) retained both the oligomeric state and binding activity of the unfused counterparts, indicating that the designed heterodimers are quite robust to fusion (Fig. S3D, S6E, S8). There are 74 different possible heterodimeric complexes that can be assembled from these fusions, each with different shapes. The majority of the fusions involve protomers of LHD274 and LHD101; fusions to LHD101 protomers alone enable the formation of 30 distinct heterodimeric complexes (Fig. S9).

Larger multicomponent hetero-oligomeric protein assemblies require subunits that can interact with more than one binding partner at the same time. To this end, we generated single chain bivalent connector proteins. Designed protomers that share the same DHR as fusion partner and have compatible termini can be simply spliced together into a single protein chain on overlapping DHR repeats (Fig. 3B). Mixing a linear connector (“B”) with its two cognate binding partners (“A” and “C”) yields a linearly arranged heterotrimer (“ABC”) in which the two terminal capping components A and C are connected through component B, but otherwise are not in direct contact with each other (Fig. 3C). We analyzed the assembly of this heterotrimer and controls by SEC (Fig. 3C), and observed stepwise assembly of the ABC heterotrimer with clear baseline separation from AB and BC heterodimers, as well as from monomeric components (Fig. 3C). Using experimentally validated linear connectors created using the above described modular splicing approach (Fig. 3D and Fig. S10A and supplementary excel file 1), we in total assembled 20 heterotrimers including one verified by negative-stain electron microscopy (nsEM) (Fig. S10B and S11). The absence of off-target complexes in these assemblies corroborates the orthogonality of the heterodimer interfaces (Fig. S12).

By employing more than one connector subunit, larger linear hetero-oligomers can be generated. We constructed and confirmed assembly of ABCA and ABCD heterotetramers, each containing two different linear connectors (B and C) and either one or two terminal caps (2xA, or A+D), an ABBA heterotetramer using a homodimeric central connector (2xB) and one terminal cap (2xA), and a negative stain EM verified heteropentamer (ABCDE) containing 3 different linear connectors and two caps (Fig. 3D, Fig. S13–14). We followed the assembly of an ABCDEF hetero-hexamer in SEC by GFP-tagging one of the components and monitoring GFP absorbance. The full assembly as well as sub-assemblies generated as controls eluted as monodisperse peaks, with elution volumes agreeing well with expected assembly sizes (Fig 3E). Negative stain EM reconstruction of the hexamer confirmed all components were present (Fig. 3E and S15A). Deviation of the experimentally observed shape from the design model likely arises from small deviations from the model in one of the components that cause a lever-arm effect (Fig. 2B).

In total, by combining the bivalent connectors with each other and with monovalent terminal caps, we constructed 36 hetero-oligomers with up to 6 different chains and confirmed their assembly by SEC and electron microscopy (Fig. 3C;3E, Fig. S10–11 and S13–15, supplementary excel file 1 (experimentally_validated_assemblies)). This number can be readily increased to 489 by including all available components (Fig. 3A, Fig. S10A and supplementary excel file 1 (all_theoretical_assemblies)). Since all fusions have structured helical linkers, the overall molecular shapes of the complexes and the spatial arrangement of individual components are well defined, which should be useful for scaffolding and other applications. Our linear assemblies resemble elongated modular multi-protein complexes found in nature (Fig. S15B), like the Cullin RING E3 Ligases (29) that mediate ubiquitin transfer by geometrically orienting the target protein and catalytic domain.

**Fig. 4.**
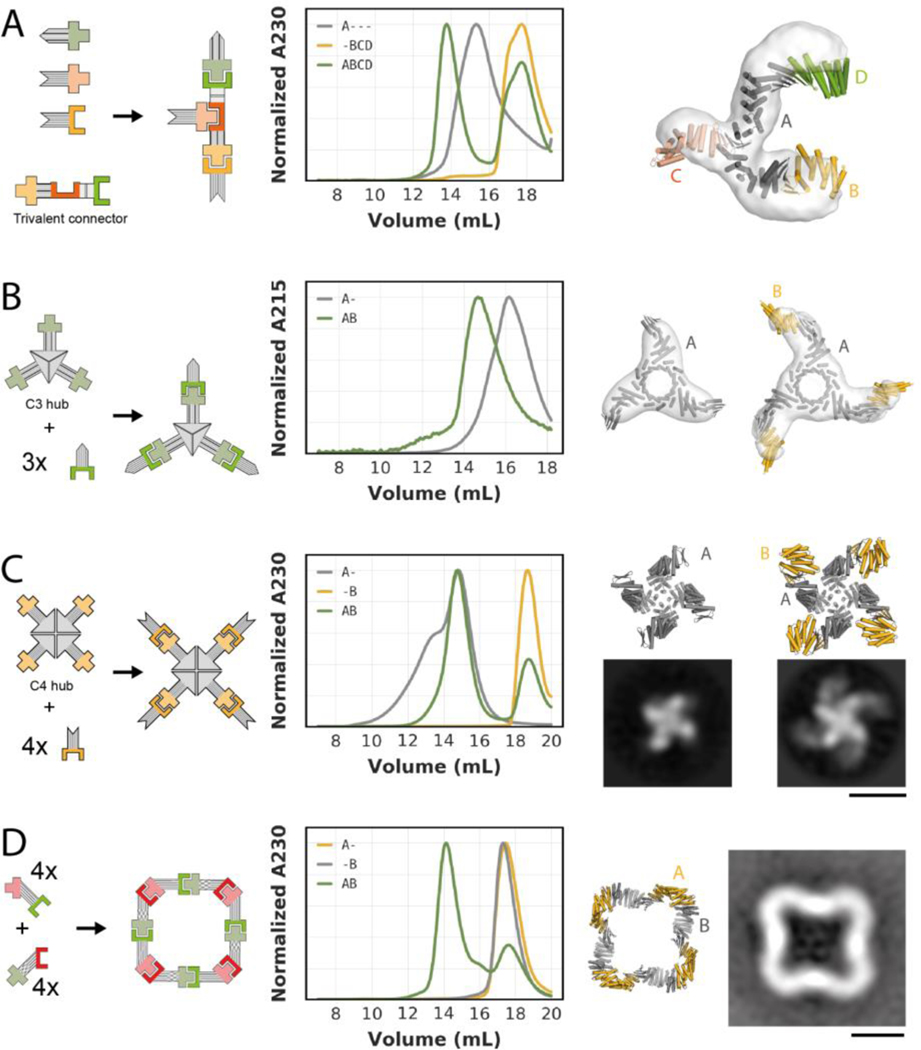
Design of branched and closed hetero-oligomeric assemblies. **(A)** Left: Schematic representation of a trivalent connector (“A” = TF10) that can bind three different binding partners (“B” = 274A53, ”C” = 317B, “D” = 101B62). Center: SEC analysis of the trivalent connector, the binding partners, and the full assembly mixture. Right: Overlay of design model (in colors) and nsEM density (light grey) of the complex formed by the trivalent connector and all three binding partners. **(B)** From left to right: : Schematic representation of a C3-symmetric “hub” presenting three copies of LHD101B; SEC analysis of the C3-symmetric “hub” without (“A-”) and with (“AB”) its cognate binding partner (“B” = 101A53); overlay of design model (dark grey) and nsEM density (light grey) of the C3-symmetric “hub”; overlay of design model (dark grey and gold) and nsEM density (light grey) of the C3-symmetric “hub” bound to three copies of its binding partner. **(C):** From left to right: : Schematic representation of a C4-symmetric “hub” presenting four copies of LHD274B; SEC analysis of the C4-symmetric “hub” without (“A-”) and with (“AB”) its cognate binding partner (274A53); design model (top) and representative nsEM class average (bottom) of the C4-symmetric “hub”; design model (top) and representative nsEM class average (bottom) of the C4-symmetric “hub” bound to 4 copies of the binding partner. **(D)** From left to right: Schematic representation of a C4-symmetric closed ring comprising two components (“A” and “B”); SEC analysis of the individual ring components (“A-” and “-B”) and the stoichiometric mixture (“AB”); design model of the C4-symmetric ring; representative nsEM class average. Scale bars: 10 nm.

We next sought to go beyond linear assemblies and build branched and closed assemblies. Trivalent connectors can be generated from heterodimers in which one protomer has both N- and C-terminal helices (LHD275A, LHD278A, LHD289A, LHD317A). Such protomers can be fused to two helical repeat proteins and spliced together with different halves of other heterodimer protomers via a common DHR repeat (Fig. 3A,B and 4A). The resulting branched trivalent connectors (“A”) are capable of binding the three cognate binding partners (“B”,”C”,”D”) simultaneously and conceptually resemble Ste5 and related scaffolding proteins that organize MAP kinase signal transduction pathways in eukaryotes (30). Through SEC analyses we verified the assembly of two different tetrameric branched ABCD complexes, each containing one trivalent branched connector bound to three terminal caps (Fig. 4A and S16). For one of these, the complex was confirmed by negative stain EM class averages and 3D reconstructions which indicate not only that all binding partners are present, but also that the shape closely matches the designed model (Fig. 4A and S16A).

A different type of branched assemblies are “star shaped” oligomers with cyclic symmetries, akin to natural assemblies formed by IgM and the Inflammasome (31, 32). Using the alignment approach described above (Fig. 3B), we fused our building blocks (Fig. 3A) to previously designed homo-oligomers (23, 33), that terminate in helical repeat proteins (Fig. 4B,C). Such fusions yield central homo oligomeric hubs (“A_n”) that can bind multiple copies of the same binding partner (“n*B”). We generated C3- and C4-symmetric “hubs” that can bind 3 or 4 copies of their binding partners, respectively (Fig. 4B,C). In both cases, the oligomeric hubs are stable and soluble in isolation and readily form the target complexes when mixed with their binding partners, as confirmed by SEC chromatography, negative stain EM class averages and 3D reconstructions (Fig. 4B,C and Fig. S17–19). For the C4-symmetric hub in the absence of its binding partner we observed an additional concentration-dependent peak on SEC (Fig. 4C, Fig. S18A, S19A), indicating formation of a higher-order complex. This is likely a dimer of C4 hubs, since the C4 hub contains the redesigned protomer LHD274B, that despite its reduced homodimerization propensity compared to parent design LHD29B still weakly homodimerizes (Fig. S5). Addition of the binding partner drives reconfiguration of this higher order assembly into the on-target octameric (A4B4) complex (Fig. 4C).

In addition to linear and branched assemblies, we designed closed symmetric two-component assemblies. Designing these presents a more complex geometric challenge, as the interaction geometry of all pairs of subunits must be compatible with a single closed three dimensional structure of the entire assembly. We used architecture-aware rigid helical fusion (7, 34) to generate two bivalent connector proteins from the crystal-verified fusions of LHD29 and LD101 (Fig 2B) that allow assembly of a perfectly closed C4-symmetric hetero-oligomeric two-component ring (Fig. 4D). Individually expressed and purified components are stable and soluble monomers in isolation, as confirmed by SEC, multi angle light scattering (MALS) and native MS (Fig. 4D, Fig. S20). Upon mixing, the components form a higher-order complex that by native MS and MALS comprises four copies of each component. Negative stain EM confirmed that this higher-order complex is similar to the designed C4 symmetric ring (Fig. 4D, Fig. S21).

**Fig. 5.**
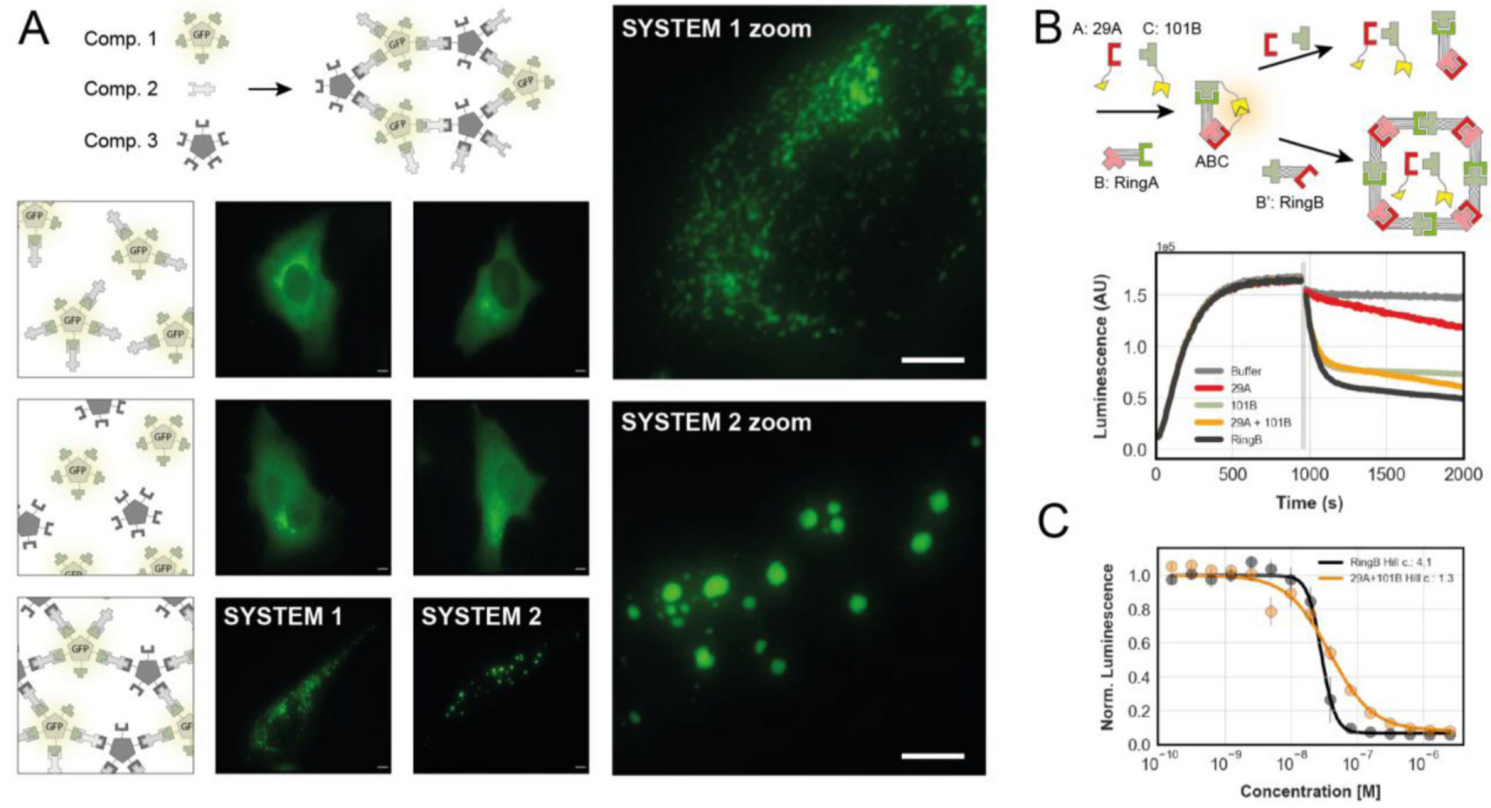
Inducible and reconfigurable assemblies. **(A)** Cross-linking of homo-pentamers by bivalent connectors in cells. Top: Schematic representation of components. Bottom: schematic representations (1st column) and fluorescence microscopy images (2nd and 3rd columns) of cells expressing different combinations of components. High affinity system 1 (2nd column) uses LHD101 and LHD275; low affinity system 2 (3rd column) uses LHD101 and LHD321. See Fig. S22 for additional control images. Scale bars: 5 μm. **(B)** Top: schematic representation of an “ABC” heterotrimer with split luciferase activity (yellow shapes) undergoing subunit exchange through addition of non-luciferase tagged components. Bottom: Real-time luminescence measurement of samples containing the mixture “ABC” shown on the top left. Grey bar indicates addition of either buffer (grey trace) or non-luciferase tagged components LHD29A and LHD101B. **(C)** Titration of either component RingB or non-luciferase tagged components LHD29A and LHD101B to the preformed ABC heterotrimer. Data fitted to the hill equation. Error bars represent sd.

To determine whether our components function as designed in living cells, and to evaluate their use in constructing conditional assemblies, we fused one heterodimer protomer to a previously designed GFP-tagged C5 homo-oligomer (7), and a second protomer of a different heterodimer to an untagged C5 homo-oligomer. Transient expression of the two constructs in HeLa cells led to a distributed and diffuse GFP signal throughout the cell (Fig. 5A, Fig. S22), suggesting that the components do not interact with each other or self associate. However, when a bivalent connector (Fig. 3B) designed to link the two homo-oligomers was also expressed, the GFP signal redistributed into discrete puncta consistent with the expected 3-component extended meshwork (Fig. 5A and Fig. S22). Notably, changing just one the two heterodimer interfaces in the assembly from a high to low affinity interface had a striking effect on the morphology of the puncta. When both interfaces had nanomolar affinity (Fig. 5A, system 1) there were many small puncta, whereas substitution with a micromolar affinity heterodimer with a more rapid dissociation rate led to large droplet-like puncta (Fig. 5A, system 2). These results show that, as designed, the components of the heterodimers are well behaved in isolation and assemble when combined in cells. The morphology differences further suggest that the ability to modulate dissociation rates and affinities of designed components could be advantageous for probing phase transitions in cells.

Because our designed building blocks are stable in solution and not kinetically trapped in off-target homo-oligomeric states, the assemblies they form can in principle reconfigure, as outlined in Figure 1A and observed for the C4-symmetric hub shown in Figure 4C. To examine reconfiguration dynamics, we constructed an ABC linear heterotrimer in which the B connector component is one of the two components of the ring shown in Figure 4D, and the A and C capping components are tagged with split luciferase fragments. In absence of B, components A and C do not interact, and luciferase activity is not reconstituted (Fig. 5B). Upon addition of B, the heterotrimer forms, resulting in luciferase activity (Fig. 5B). Addition of the other ring component (B’) to the preformed ABC trimer leads to a rapid decrease in luciferase activity, consistent with disassembly of the trimer and formation of the ring (Fig. 5B, Fig. S23A–C). Because ring formation is cooperative due to the additional interactions made upon ring closure, we reasoned that the concentration dependence of ABC trimer dissociation would be steeper upon addition of B’ than with untagged A and C. To investigate this, we titrated B’ and non luciferase tagged variants of A and C into the preformed trimer. There was a steep concentration dependence to the loss in luciferase signal upon addition of B’ with a Hill-coefficient of 4.1, (Fig. 5C and Fig. S23D) consistent with the cooperative formation of a symmetrically closed ring (4B4B’). In contrast, the loss of luciferase signal upon addition of nontagged A and C had a Hill coefficient close to 1, as expected for formation of a non-cooperative linear assembly (Fig. 5C and Fig. S23D). In both cases, reconfiguration occurred on the several minute time scale (Fig. S23B–C). We also observed reconfiguration of heterotrimers using SEC and BLI (Fig. S24). This behaviour, although common in naturally evolved protein complexes, has been difficult to achieve by design, as it requires that the individual components not self-associate on their own. Our design principles pave the way for design of functions requiring reconfigurable multiprotein complexes.

## Discussion

Our implicit negative design principles enable the de novo design of heterodimer pairs for which the individual protomers are stable in solution and readily form their target heterodimeric complexes upon mixing, unlike previously designed assemblies. Rigid fusion of components through structured helical linkers enables the design of higher order asymmetric multiprotein complexes in which individual subunits have well defined positions relative to each other. While rigidly fused building blocks may still exhibit flexibility (molecular breathing), fusion with structured connectors allows more control of subunit orientation than can be achieved by flexible linker fusion, and enables fine tuning of protein complex geometries. Because of the small sizes of our unfused protomers (between 7 and 15 kDa without DHR or tags), complexes can readily be functionalized through genetic fusion of subunits with proteins of interest. Our bivalent or trivalent connectors can then be used to colocalize and geometrically position two or three such target protein fusions, respectively, and our symmetric hubs can be used to colocalize and position multiple copies of the same target fusion. Due to the modularity of our system, the same set of target fusions can be arranged in multiple different arrangements with adjustable distances, angles, and copy numbers by simply using different components (Fig. S25). Because of the solubility and stability of the designs in isolation, complexes can be assembled stepwise (see for example Fig. 5A). The asymmetric complexes generated with our components will in general have low assembly cooperativity so the fraction of fully assembled complex will be sensitive to the concentrations of the individual components over a broad range, enabling subunit exchange and complex reconfiguration in response to signal inputs for synthetic biology and other applications. Since the thermodynamics and kinetics of our designed interfaces are not altered by fusion, the fraction of full assemblies and subassemblies, and assembly dynamics, can in principle be predicted based on the properties of the individual interfaces ( Fig. S23A). We expect that the design approach and components presented here will lead to a new generation of reconfigurable protein assemblies for a wide range of applications--for example intracellular control for synthetic biology, design of protein logic gates, reprogramming cells from the outside by arraying receptor binding modules with specific geometries, processive multi enzyme complexes, and designed molecular machines.

## Materials and Methods

### Protein design

#### Docking procedure

As scaffolds for generating edge-strand heterodimers we used mixed alpha/beta proteins designed by citizen scientist (22) and variants of the fold-it scaffolds that were either expanded with additional helices (see backbone generation methods), and/or fused to de novo helical repeat (DHR) proteins (28). Edgestrand docking was performed as described previously (19). Exposed edgestrands suitable for docking were identified by calculating the solvent accessible surface area of beta sheet backbone atoms in all the scaffolds used in the docking procedure. Next, the c-alpha atoms of each strand of short 2 stranded parallel and antiparallel beta sheet motifs were aligned to the exposed edge strand yielding an aligned clashing strand and free dock strand. After removal after the aligned clashing strand, the docked strand was trimmed at N and/or C terminus in order to remove potential clashes and subsequently minimized using Rosetta FastRelax (35) to optimize backbone to backbone hydrogen bonds. Docks failing a specified threshold value (typically −4 using ref2015) for the backbone hydrogen bond scoreterm in Rosetta (hbond_lr_bb) were discarded. The minimized docked strands were next geometrically matched to the scaffold library using the MotifGraftMover to create a docked protein-protein complex (36).

#### Interface design

The interface residues of the docked heterodimer complexes were optimized using Rosetta combinatorial sequence (37–40) design using “ref2015”, “beta_nov16” or “beta_genpot” as scorefunctions (41). The interface polarity of the docked heterodimer complexes were fine tuned in several ways (see supplement for description of design xml’s). First, the HBNetMover (11) was used to install explicit hydrogen bond networks containing at least 3 hydrogen bonds across the interface. Later design rounds consisted of two seperate interface sequence optimization steps. First interface residues were optimized without compositional constraints yielding a substantial number of hydrophobic interactions in the interface. The best designs were subsequently selected and hydrophobic residue pairs with the lowest Rosetta energy interactions across the interface were stored as a seed hydrophobic interaction hotspot. In a second round, a polar interaction network was designed around the fixed hydrophobic hotspot interaction using compositional constraints that favor polar interactions (27). Designs were filtered on interface properties such as binding energy, buried surface area, shape complementarity, degree of packing, and presence of unsatisfied buried polar atoms. A final selection was made by visual inspection of models.

#### Homodimer self-docking

In later design rounds the propensity for homodimerization was explicitly assessed *in silico.* Each individual chain of a heterodimer, was docked onto itself via edge-strand docking (19) (see also Docking procedure section methods). This creates a set of disembodied strands that pair with the scaffold edge strand that also participates in the heterodimeric complex. Homodimer docks were generated by aligning the heterodimerizing edge strand of a second copy of the scaffold back onto the disembodied docked strand (see fig. S7A). Docks with different beta register offsets and orientations (parallel/anti-parallel) were created. Docks were next converted to polyglycine and clash checked. Docks where the repulsive Rosetta scoreterm (fa_rep) was higher than 250 (scorefunction ref2015) were discarded (i.e. no homodimer possible). Surviving docks were converted to full atom models and minimized using FastRelax (35) followed by scoring/assessing of homodimer interface metrics such as binding energy, buried surface area, shape complementarity, degree of packing, and presence of unsatisfied buried polar atoms.

#### Backbone generation and scaffold design

*De novo* designed protein scaffolds created by fold-it players (22) were expanded with C-terminal polyvaline helices using blueprint based backbone generation (24, 25). The amino acid identities of the newly built helices and their surrounding region were optimized using Rosetta combinatorial sequence designs using a flexible backbone. The resulting models were folded *in silico* using Rosetta folding simulations and trajectories that converged to the designed model structure without off-target minima were selected for rigid fusion and heterodimer design.

#### Design of rigid fusions

To generate rigid fusions of scaffolds or heterodimers to DHRs we adapted the HFuse pipeline (7, 23): Fusion junctions were designed using the Fastdesign mover allowing backbone movement, and additional filters were included to ensure sufficient contact between DHR and scaffold/heterodimer. When fusing to heterodimers, an additional filter was employed to prevent additional contacts between the DHR and the other protomer of the dimer. Bivalent connectors were generated by aligning two proteins that share the same DHR along their shared helical repeats, and subsequently splicing together the sequences. To build the C3-symmetric “hub”, we used a previously published 12x toroid crystal structure (33). The starting structure was relaxed, Z axis aligned, and cut into three C3 symmetric chains. Then the HFuse software (7, 23) was used to sample DHR fusions to the exposed helical C-termini, and the newly created interfaces were redesigned using RosettaScripts. For the C4 symmetric hub, we used a previously published C4-symmetric homooligomer that already containe a n-terminal DHR. For both hubs, matching DHR fusions of heterodimer protomers we then used the same align and splice approach as for the bivalent connectors.

#### Design of C4 rings

Using the relaxed crystal structures of LHD29 and LHD101 fused to their respective DHRs, the WORMS software (7, 9, 34) was used to fuse the two hetero-dimers into cyclic symmetrical rings. As one construct has exposed N-termini and the other has exposed C-termini, they were able to be fused head to tail without introduction of further building blocks. Briefly, the first 3 repeats of each repeat protein was allowed to be sampled as fusion points to ensure that the heterodimer interface was not altered. Following fusion into cyclic structures, fixed backbone junction design was applied to the new fusion point using RosettaScripts (39), optimizing for shape complementarity (42). One design from each symmetry: C3, C4, C5, and C6 were selected for experimental testing.

#### Protein expression and purification

Synthetic genes encoding designed proteins and their variants were purchased from Genscript or Integrated DNA technologies (IDT). Bicistronic genes were ordered in pET29b with the first cistron being either without tag or with an N-terminal sfGFP tag followed by the intercistronic sequence TAAAGAAGGAGATATCATATG. The second cistron was tagged with a polyhistidine His6x tag at the C-terminus. Plasmids encoding the individual protomers were ordered in pET29b either with or without Avi-Tag, with an N-terminal polyhistidine His6x tag followed by a TEV cleavage site, N-terminal polyhistidine His6x tag followed by a snac cleavage site or C-terminal polyhistidine His6x tag preceded by a snac tag (see supplementary spreadsheet for detailed construct information). Proteins were expressed in BL21 LEMO E.coli cells by autoinduction using TBII media (Mpbio) supplemented with 50×5052, 20 mM MgSO4 and trace metal mix, or in almost TB media containing 12 g peptone and 24 g yeast extract per liter supplement with 50×5052, 20 mM MgSO4, trace metal mix and 10x phosphate buffer. Proteins were expressed under antibiotics selection at 37 degrees overnight or at 18 degrees for 24h after initial growth for 6–8h at 37 degrees. Cells were harvested by centrifugation at 4000x g and lysed by sonication after resuspension of the cells in lysis buffer (100 mM Tris pH 8.0, 200 mM NaCl, 50 mM Imidazole pH 8.0) containing protease inhibitors (Thermo Scientific) and Bovine pancreas DNaseI (Sigma-Aldrich). Proteins were purified by Immobilized Metal Affinity Chromatography. Cleared lysates were incubated with 2–4ml nickel NTA beads (Qiagen) for 20–40 minutes before washing beads with 5–10 column volumes of lysis buffer, 5–10 column volumes of high salt buffer (10 mM Tris pH 8.0, 1 M NaCl) and 5–10 column volumes of lysis buffer. Proteins were eluted with 10 ml of elution buffer (20 mM Tris pH 8.0, 100 mM NaCl, 500 mM Imidazole pH 8.0).

Designs were finally polished using size exclusion chromatography (SEC) on either Superdex 200 Increase 10/300GL or Superdex 75 Increase 10/300GL columns (GE Healthcare) using 20 mM Tris pH 8.0, 100 mM NaCl or 20 mM Tris pH 8.0, 300 mM NaCl. Cyclic assemblies of C3 and C4 symmetries were purified using a Superose 6 increase 10/300GL (GE Healthcare). The two component C4 rings were SEC purified in 25 mM Tris pH 8.0, 300 mM NaCl. Peak fractions were verified by SDS-PAGE and LC/MS and stored at concentrations between 0.5–10 mg/ml at 4 degrees or flash frozen in liquid nitrogen for storage at −80. Designs that precipitated at low concentration upon storage at 4 degrees could in general be salvaged by increasing the salt concentration to 300–500 mM NaCl.

For structural studies, designs with a polyhistidine tag and TEV recognition site were cleaved using TEV protease (his6-TEV). TEV cleavage was performed in a buffer containing 20 mM Tris pH 8.0, 100 mM NaCl and 1 mM TCEP using 1% (w/w) his6-TEV and allowed to proceed o/n at room temperature. Uncleaved protein and his6-TEV were separated from cleaved protein using IMAC followed by SEC. Designs carrying a C-terminal SNAC-polyhistine tag (GGSHHWGS(...)HHHHHH) were cleaved chemically via on-bead nickel assisted cleavage (43): nickel bound designs were washed with 10CV of lysis buffer followed by 5CV of 20 mM Tris pH 8.0, 100 mM NaCl. Proteins were subsequently washed with 5CV of SNAC buffer (100 mM CHES, 100 mM Acetone oxime, 100 mM NaCl, pH 8.6). Beads were next incubated with 5CV SNAC buffer + 2 mM NiCl_2_ for more than 12 hours at room temperature on a shaking platform to allow cleavage to take place. Next, the flow through containing cleaved protein was collected. The flow throughs of two additional washes (SNAC buffer/SNACbuffer+50 mM Imidazole) of 3–5CV were also collected to harvest any remaining weakly bound protein. Cleaved proteins were finally purified by SEC.

For mammalian cell expression, synthetic genes encoding designed proteins were purchased from Genscript and cloned into mammalian expression vectors. LHD101B-C5 was cloned into the KpnI/XbaI site of pCDNA3.1+N-eGFP in frame with eGFP. Both LHD275B_53_0_LHD101A and LHD321B_53_LHD101A were cloned into the NheI/XbaI site of pCDNA3.1+C-HA. LHD275A-C5 and LHD321A-C5 were cloned into KpnI/XbaI site of pCDNA3.1+N-HA.

#### Cell culture and transient transfections

HeLa cells (ATCC CCL-2) were cultured in DMEM (Gibco) that was supplemented with 1 mM L-glutamine (Gibco), 4.5 g/L D-Glucose (Gibco), 10% fetal bovine serum (FBS) and (1x) non-essential amino acids (Gibco). Cells were cultured at 37°C and 5% CO2 and passaged twice per week. To passage, cells were dissociated using 0.05% Trypsin EDTA (Gibco) and split 1:5 or 1:10 into a new TC-treated T75 flask (Thermo Scientific ref 156499).

HeLa cells were plated at 20,000 cells per well in a Cellview Cell Culture Slide, PS, 75/25mm, Glass Bottom, 10 Compartments, TC-treated (Greiner Bio-One ref 543079). 24 hours later, cells were transiently transfected at a concentration of 187.5ng total DNA per well and 1ug/uL PEI-MAX (Polyscience) mixed with Opti-MEM medium (Gibco). Transfected cells were incubated at 37°C and 5% CO2 for 24–36 hours before being imaged.

#### Fluorescence microscopy and image processing

3D images were acquired with a commercial OMX-SR system (GE Healthcare). A 488nm Toptica diode laser was used for excitation. Emission was collected on a PCO.edge sCMOS cameras using an Olympus 60× 1.42NA PlanApochromat oil immersion lens. 1024×1024 images (pixel size 6.5 μm) were captured with no binning. Acquisition was controlled with AcquireSR Acquisition control software. Z-stacks were collected with a step size of 500 nm and 15 slices per image. Images were deconvolved with an enhanced ratio using SoftWoRx 7.0.0 (GE Healthcare). Cell images were sum projected using Fiji v2.1.0. Scale bars equal 5 microns.

#### Enzymatic protein biotinylation

Avi-tagged (GLNDIFEAQKIEWHE, see supplement) proteins were purified as described above. The BirA500 (Avidity, LLC) biotinylation kit was used to biotinylate 840 uL of protein from the IMAC elution in a 1200 uL (final volume) reaction according to the manufacturer’ protocol. Reactions were incubated at 4 degrees C o/n and purified using size exclusion chromatography on a Superdex 200 10/300 Increase GL (GE Healthcare) or S75 10/300 Increase GL (GE Healthcare) in SEC buffer (20 mM Tris pH 8.0, 100 mM NaCl).

#### Biolayer interferometry

Biolayer interferometry experiments were performed on an OctetRED96 BLI system (ForteBio, Menlo Park, CA). Streptavidin coated biosensors were first equilibrated for at least 10 minutes in Octet buffer (10 mM HEPES pH 7.4, 150 mM NaCl, 3 mM EDTA, 0.05% Surfactant P20) supplemented with 1 mg/ml Bovine Serum Albumin (SigmaAldrich). Enzymatically biotinylated designs were immobilized onto the biosensors by dipping the biosensors into a solution with 10–50 nM protein for 30–120 s. This was followed by dipping in fresh octet buffer to establish a baseline for 120 s. Titration experiments were performed at 25 °C while rotating at 1,000 r.p.m. Association of designs was allowed by dipping biosensors in solutions containing designed protein diluted in octet buffer until equilibrium was approached followed by dissociation by dipping the biosensors into fresh buffer solution in order to monitor the dissociation kinetics. Steady-state and global kinetic fits were performed using the manufacturer’s software (Data Analysis 9.1) assuming a 1:1 binding model.

#### SEC binding assays

Complexes and individual components were diluted in 20 mM Tris pH 8.0, 100 mM NaCl. After o/n equilibration of the mixtures at room temperature or 4 degrees C, 500 ul of sample was injected onto a Superdex 200 10/300 increase GL (dimers, linear assemblies) or Superose 6 increase 10/300 GL (symmetric assemblies) (all columns from GE healthcare) using the absorbance at 230 nm or 473 nm (for GFP tagged components) as read-out. Dimers were mixed at monomer concentrations of 5 μM or higher. Trimer and ABCD tetramer mixtures contained 5 μM of the bivalent connector, and 7.5 μM of each terminal cap (lower absolute concentrations with the same ratios were used for some trimers). ABCA tetramer mixtures contained 5 μM per bivalent connector and 15 μM terminal cap. The hexamer mixture contained 3 μM of components C and D, 3.6 μM of B and E, and 4.4 μM of A and F. The branched assembly shown in Figure 4A contained 2.8 μM of the trivalent connector and 4 μM of each cap. For the exchange experiment shown in Fig. S24A, the ABC trimer was preincubated at concentrations of 6 μM B and 9 μM each of A and C. C’ was then added to reach a final concentration of 2 μM B, 3 μM each of A and C, and 6 μM C’.

#### Native mass spectrometry

Sample purity, integrity, and oligomeric state was analyzed by on-line buffer exchange MS in 200 mM ammonium acetate using a Vanquish ultra-high performance liquid chromatography system coupled to a Q Exactive ultra-high mass range Orbitrap mass spectrometer (Thermo Fisher Scientific). A self-packed buffer exchange column was used (P6 polyacrylamide gel, BioRad) (44). The recorded mass spectra were deconvolved with UniDec version 4.2+ (45).

#### Crystal structure determination

For all structures, starting phases were obtained by molecular replacement using Phaser (46). Diffraction images were integrated using XDS (47) or HKL2000 (48) and merged/scaled using Aimless (49). Structures were refined in Phenix (50) using phenix.autobuild and phenix.refine or Refmac (51). Model building was performed using COOT (52).

Proteins were crystallized using the vapor diffusion method at room temperature. LHD29 crystals grew in 0.2M Sodium Iodide, 20% PEG3350, LHD29A53/B53 crystals in E5 and LHD101A53/B4 crystals in 2.4M Sodium Malonate pH 7.0. Crystals were harvested and cryoprotected using 20% PEG200 for LHD29, 20% PEG400 for LHD29A53/B53 and 20% glycerol for LHD101A53/B4 before data was collected at the Advanced Light Source (Berkeley, USA). The structures were solved by molecular replacement using either computationally designed models of individual chains A or B or the full heterodimer complex as search models.

The RMSD, TMscore and LDDT metrics between the designed models and corresponding crystal structures were calculated as described previously (53, 54). Protein structure graphics were prepared using PyMOL (Schrödinger).

#### Electron microscopy

SEC peak fractions were concentrated prior to negative stain EM screening. Samples were then immediately diluted 5 to 150 times in TBS buffer (25 mM Tris pH 8.0, 25 mM NaCl) depending on sample concentration. A final volume of 5 μL was applied to negatively glow discharged, carbon-coated 400-mesh copper grids (01844-F, TedPella,Inc.), then washed with Milli-Q Water and stained using 0.75% uranyl formate as previously described (55). Air-dried grids were imaged on a FEI Talos L120C TEM (FEI Thermo Scientific, Hillsboro, OR) equipped with a 4K × 4K Gatan OneView camera at a magnification of 57,000x and pixel size of 2.51. Micrographs were imported into CisTEM software or cryoSPARC software and a circular blob picker was used to select particles which were then subjected to 2D classification. Ab initio reconstruction and homogeneous refinement in Cn symmetry were used to generate 3D electron density maps (56, 57).

#### Constructs for Luciferase assays

Split luciferase reporter constructs were ordered as synthetic genes from Genscript. Each design was N-terminally fused to a sfGFP (for protein quantification in lysate), and C-terminally fused to either smBiT or lgBiT of the split luciferase components. A Strep-tag was included at the N-terminus for purification, and a GS-linker was inserted between the design and the split luciferase component.

#### Expression for multiplexed Luciferase assay

Plasmids were transformed into Lemo21(DE3) cells (New England Biolabs), and grown in 96 deepwell plates overnight at 37 °C in 1 mL of LB containing 50 ug/mL of kanamycin sulfate. The next day, 100 uL of overnight cultures were used to inoculate 96 deepwell plates containing 900 uL of TBII medium (MP Biomedicals) with 50 ug/mL of kanamycin sulfate, and the cultures were grown for 2 h at 37 °C before induction with 0.1 mM IPTG. Protein expression was carried out at 37 °C for 4 h before the cells were harvested by centrifugation (4,000 x g, 5 min). Cell pellets were resuspended in 100 uL of lysis buffer (10 mM sodium phosphate, 150 mM NaCl, pH 7.4, 1 mg/mL lysozyme, 0.1 mg/mL DNAse I, 5 mM MgCl_2_, 1 tablet/50 mL of cOmplete protease inhibitor (Roche), 0.05% v/v Tween 20), and cell were lysed by performing three freeze/thaw cycles (1 h incubations at 37 °C followed by freezing at −80 °C). The lysate was cleared by centrifugation (4,000 x g, 20 min), and the soluble fraction transferred to a 96 well assay plate (Corning, cat #3991). Concentrations of the constructs in soluble lysate were determined by sfGFP fluorescence using a calibration curve.

#### Lysate production for multiplexed Luciferase assay

Neutral lysate for preparing serial dilutions was prepared by transforming Lemo21(DE3) with the pUC19 plasmid. Transformations were used to inoculate small overnight cultures, which were used to inoculate 0.5 L TBII cultures (all cultures contained 50 ug/mL of carbenicillin). Cells were grown for 24 h at 37 °C before being harvested. Pellets were resuspended in the same lysis buffer, followed by sonication. The lysate density was adjusted with lysis buffer to have its OD280 matching pUC19 control wells from the 96 well expression plate.

#### Expression and purification of Luciferase constructs

Plasmids were transformed into Lemo21 (DE3) cells, and used directly to inoculate 50 mL of auto-induction media (TBII supplemented with 0.5 % w/v glucose, 0.05% w/v glycerol, 0.2% w/v lactose monohydrate, and 2 mM MgSO_4_, 50 ug/mL kanamycin sulfate). The cultures were incubated at 37 °C for 20–24 h, before harvesting the cells by centrifugation (4,000 x g, 5 min). Cells were resuspended in 10 mL of lysis buffer (100 mM Tris, 150 mM NaCl, pH 8, 0.1 mg/mL lysozyme, 0.01 mg/mL DNAse I, 1 mM PMSF) and lysed by sonication. The insoluble fraction was cleared by centrifugation (16,000 x g for 45 min), and the proteins were purified from the soluble fraction by affinity chromatography using Strep-Tactin XT Superflow High-Capacity resin (IBA Lifesciences). Elutions were performed with 100 mM Tris, 150 mM NaCl, 50 mM biotin, pH 8, and the proteins were further purified by size-exclusion chromatography using a Superdex 200 10/300 increase column equilibrated with 20 mM sodium phosphate, 100 mM NaCl, pH 7.4, 0.05% v/v Tween 20.

#### Luciferase Binding assays

All assays were performed in 20 mM sodium phosphate, 100 mM NaCl, pH 7.4, 0.05% v/v Tween 20. Depending on the source of the protein used in the assay (purified components or lysate), soluble lysate components were also present. Reactions were assembled in 96 well plates (Corning, cat #3686) in the presence of Nano-Glo substrate (Promega, cat. #N1130) diluted 100x or 500x for kinetics and endpoint measurements respectively, and the luminescence signal was recorded on a Synergy Neo2 plate reader (BioTek).

Kinetic binding assays were performed under pseudo first-order conditions, with the final concentration of one protein at 1 nM and the other at 10 nM. Stock solutions were mixed in a 1:1 volume ratio in the presence of substrate, and the dead-time between mixing and starting the measurement (typically 15–30 s) was added during data-processing. For long kinetic measurements (Fig. S6A), the proteins were pre-mixed, and kept in a sealed tube at room temperature over the course of the experiment. Aliquots were taken at regular intervals, mixed with substrate, and immediately recorded. All kinetic measurements were fitted to a single exponential decay function:

$$S=A^{*}exp\left(-k_{obs}*t\right)+B$$

where *t* is time (the independent variable), *S* is the observed luminescence signal (the dependent variable), and the fitted parameters are: *A* the amplitude, *k*_obs_ the observed rate constant, and *B* the endpoint luminescence.

Equilibrium binding assays were performed with one component kept constant at 1 nM while titrating the other protein. Serial dilutions curves were prepared over 12 points, with a ¼ dilution factor between each step. The concentration of protein in the soluble lysate provided the highest concentration point of the curve. To avoid serial dilution of the other lysate components, all stocks were prepared with neutral lysate. The assembled plates were incubated overnight at room temperature before adding substrate and immediately measuring luminescence. The data was fitted to the following equation to obtain *K*_d_ values:

$$S=S_{0}+S_{1}*f_{AB}+a_{2}*B_{T}*S_{2}$$

$$f_{AB}=\left(A_{T}+B_{T}+K_{d}-{\left(A_{T}+B_{T}+K_{d}\right)}^{2}-4A_{T}B_{T}\right)/\left(2A_{T}\right)$$

where *A*_*T*_ and *B*_T_ are the total concentrations of each species (the independent variables, *A*_*T*_ = 1 nM, *B*_*T*_ is the titrated species), and *S* is the observed signal (the dependent variable). The fitted parameters are: *S*_*0*_ the pre-saturation baseline, *S*_*1*_ the post-saturation baseline, *a*_*2*_ and *S*_*2*_ the correction terms, and *K*_*d*_ the equilibrium dissociation constant.

Ternary complex equilibrium binding experiments were performed with pure protein, using the concentration indicated in Fig. S23 for the constant components, and titratring B. After assembly, the plates were incubated overnight before adding substrate and immediately measuring luminescence.

Ternary complex reconfiguration kinetics (Fig. 5B and fig. S23) were measured with pure proteins. Components A (1 nM) and C (100 nM) were briefly pre-incubated in the presence of substrate (1/500 dilution), before adding component B (50 nM) to start the reaction. Once the association reactions were complete, the assay plate was briefly taken out of the plate reader, out-competing protein(s), D, (100 nM each in Fig. 5B and Fig. 23B and 1000 nM each in Fig. S23C) were added to the reactions, and data acquisition was resumed.

Ternary complex thermodynamic out-competitions (Fig. 5C and Fig. S23D) were measured with purified proteins. Final concentrations of components A-smBiT, B and C-lgBiT were 1, 50, and 100 nM final respectively. The out-competitor(s) (B’, or untagged A+C) were titrated from 10 uM down to about 1 pM over 24 points, with a ½ dilution factor between each step. Reactions were incubated at room temperature for 2–5 h before adding substrate (1/500 dilution) and measuring luminescence. The averages of four experiments were fitted to the Hill equation:

$$S=S_{0}+\left(S_{1}-S_{0}\right)/\left(1+{\left(K/L\right)}^{n}\right)$$

where *L* is the total concentration of the out-competitor(s) (the independent variable), and *S* is the observed signal (the dependent variable). The fitted parameters are: *S*_*0*_ the pre-saturation baseline, *S*_*1*_ the post-saturation baseline, *K* the transition midpoint, and *n* the Hill coefficient.

#### Simulation of ternary complex

Systems of ordinary differential equations describing the kinetics of interactions between the species involved in the formation of the ternary complex (Fig. S23A) were numerically integrated using integrate.odeint() as implemented in Scipy (version 1.6.3). Steady-state values were used to determine the distribution of species at thermodynamic equilibrium.

The ternary system is composed of the following species: A, B, C, AB, BC, ABC. The following set of equations was used to describe the system:

$$\text{d}[\text{A}]/\text{dt}=-k_{1}[\text{A}][\text{B}]+k_{-1}[\text{AB}]-k_{1}[\text{A}][\text{BC}]+k_{-1}[\text{ABC}]$$

$$\text{d}[\text{B}]/\text{dt}=-k_{1}[\text{A}][\text{B}]+k_{-1}[\text{AB}]-k_{2}[\text{B}][\text{C}]+k_{-2}[\text{BC}]$$

$$\text{d}[\text{C}]/\text{dt}=-k2[\text{B}][\text{C}]+k_{-2}[\text{BC}]-k_{2}[\text{AB}][\text{C}]+k_{-2}[\text{ABC}]$$

$$\text{d}[\text{AB}]/\text{dt}=k_{1}[\text{A}][\text{B}]-k_{-1}[\text{AB}]+k_{-2}[\text{ABC}]-k_{2}[\text{AB}][\text{C}]$$

$$\text{d}[\text{BC}]/\text{dt}=k_{2}[\text{B}][\text{C}]-k_{-2}[\text{BC}]+k_{-1}[\text{ABC}]-k_{1}[\text{A}][\text{BC}]$$

$$\text{d}[\text{ABC}]/\text{dt}=k_{1}[\text{A}][\text{BC}]-k_{1}[\text{ABC}]+k_{2}[\text{AB}][\text{C}]-k_{-2}[\text{ABC}]$$

where *k*_i_ describe bimolecular association rate constants and *k*_−i_ represent unimolecular dissociation rate constants. *K*_1_=_k−1_/ *k*_1_, and *K*_2_=*k*_−2_ / *k*_2_ describe the affinity of the A:B and B:C interfaces respectively.

## Supplementary Material

supp

Data S2

Data S1
