# Supplementary material for: Reconfigurable asymmetric protein assemblies through implicit negative design
Source: Science. Author manuscript; Available in PMC 2023 Jan 27. (PMC9881579; doi:10.1126/science.abj7662)
Supplement: supp [file NIHMS1843281-supplement-supp.docx]

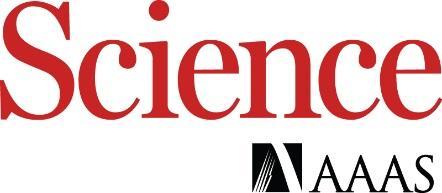


Supplementary Materials for

Reconfigurable asymmetric protein assemblies through implicit negative design

Danny D. Sahtoe^1,2,3,4^, Florian Praetorius^1,2,4^, Alexis Courbet^1,2,3^, Yang Hsia^1,2^, Basile I.M. Wicky^1,2^, Natasha I. Edman^1,2,5,6^, Lauren M. Miller^1,2^, Bart J. R. Timmermans^1,2^, Justin Decarreau^1,2^, Hana M. Morris^1,2^, Alex Kang^1,2^, Asim K. Bera^1,2^, David Baker^1,2,3,*^

^1^Department of Biochemistry, University of Washington, Seattle, WA 98195

^2^Institute for Protein Design, University of Washington, Seattle, WA 98195

^3^HHMI, University of Washington, Seattle, WA 98195

^4^Equal contribution

^5^Molecular and Cellular Biology Graduate Program, University of Washington, Seattle, WA, USA.

^6^Medical Scientist Training Program, University of Washington, Seattle, WA, USA

^*^Corresponding author

Correspondence to: [dabaker@uw.edu](mailto:dabaker@uw.edu)

**This PDF file includes:**

Figs. S1 to S25

Tables S1 to S5

Captions for Data S1 to S2

**Other Supplementary Materials for this manuscript include the following:**

Data S1 to S2: DataS1_components_and_assemblies.xlsx, DataS2_pdbs_and_scripts.gz


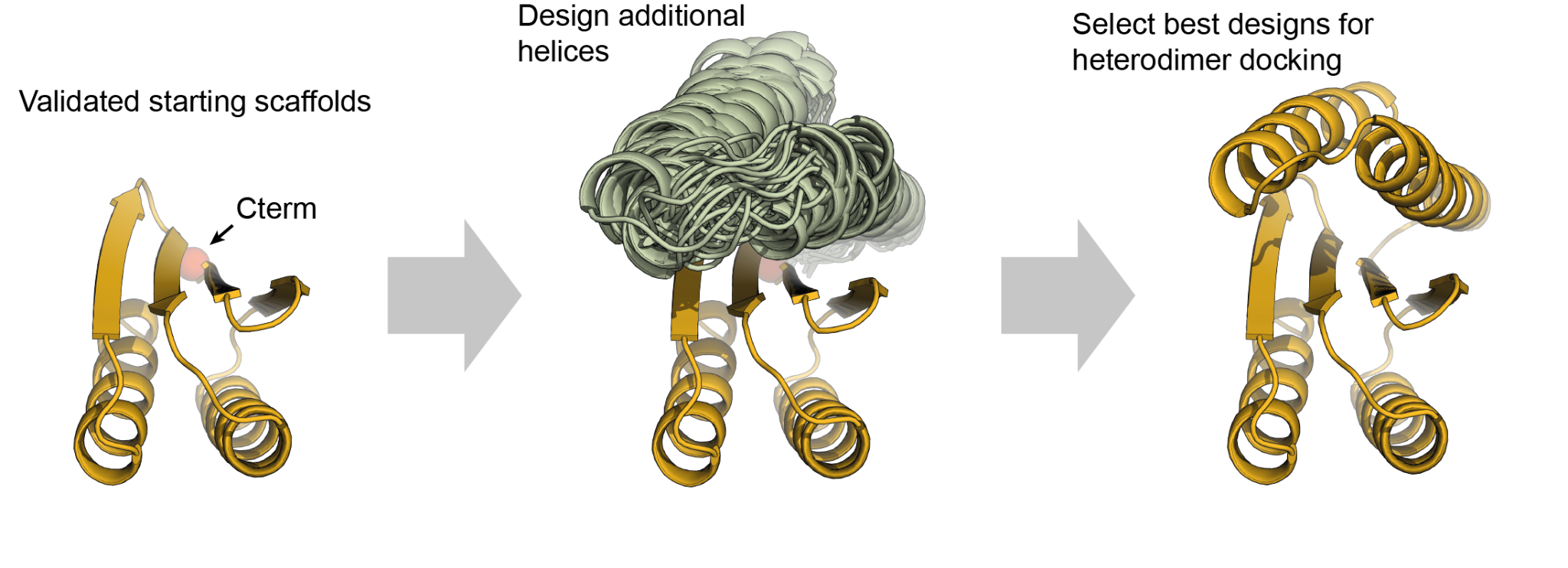


**Figure S1. Modification of Fold-it scaffolds.** Fold-it scaffold 2003333_0006 (left) was expanded with 2 additional helices (middle) on its C-terminus via blueprint-based backbone generation. After backbone generation, the scaffold sequence was designed and the best scaffolds were selected (right)  based on per residue rosetta energy and core packing.


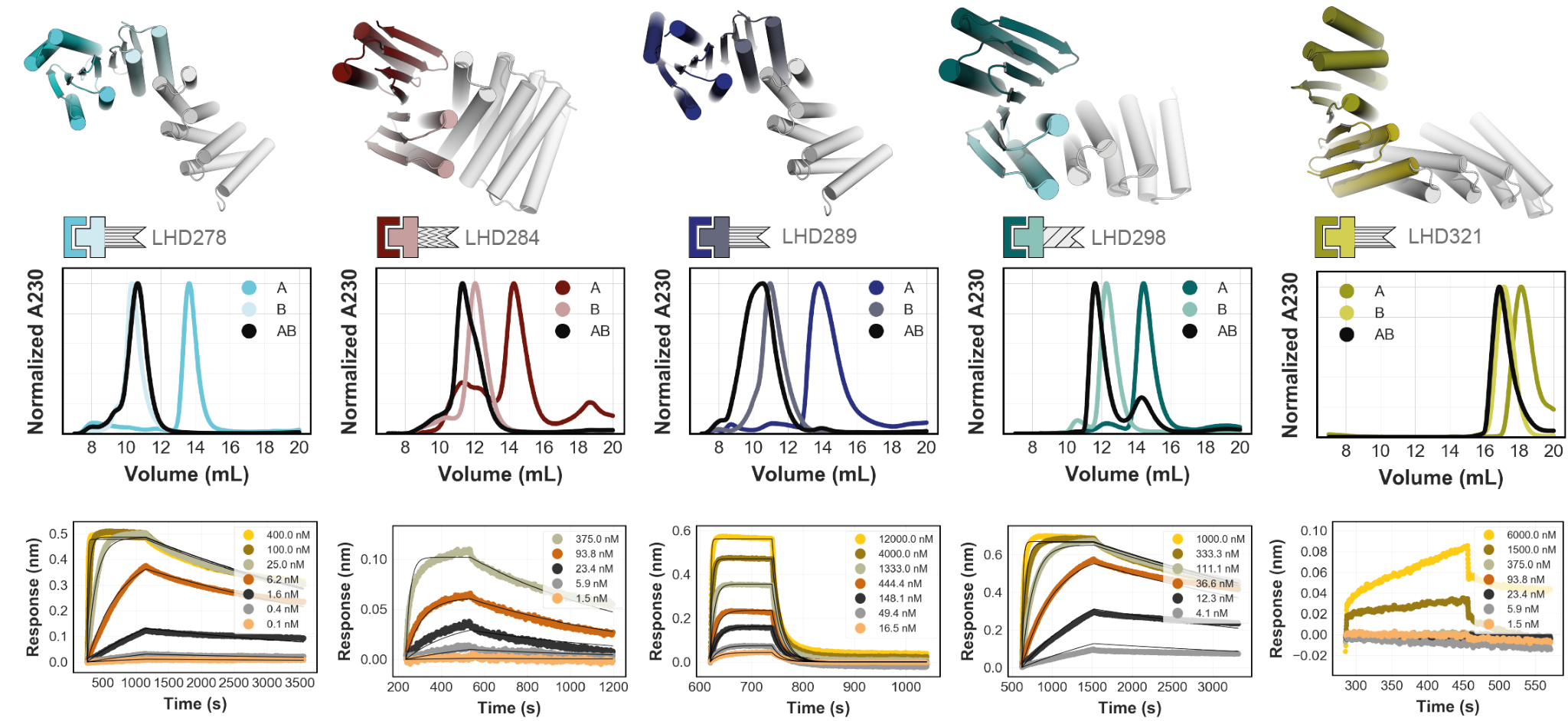


**Figure S2. Characterization LHD binding in vitro. A:** Top row, heterodimer design models. Coloring of heterodimer schematics is maintained throughout the paper. Middle row, SEC binding experiments performed on a superdex 75 column. Bottom row, biolayer interferometry kinetic binding  traces.


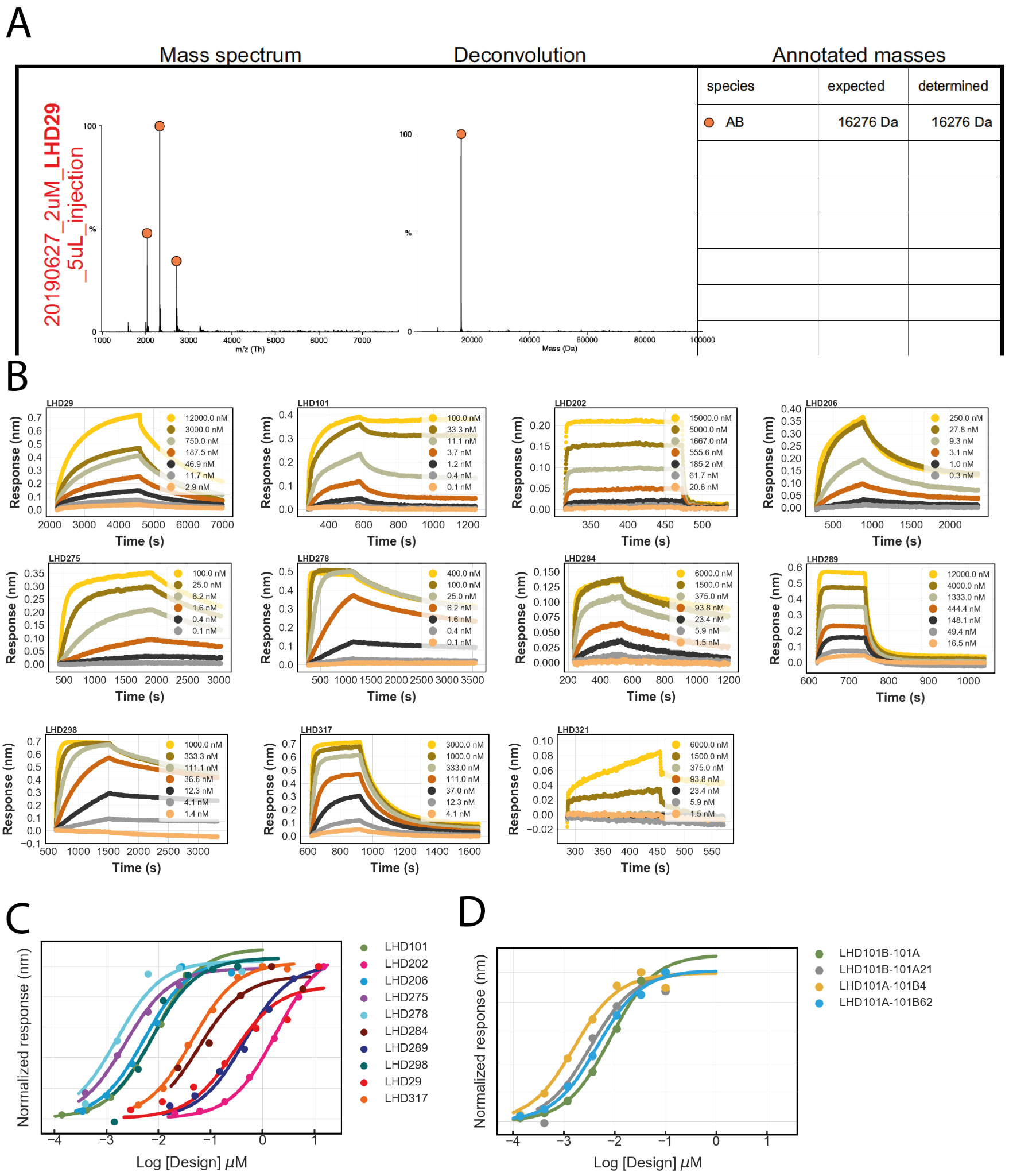


**Figure S3. Characterization LHD binding in vitro. A:** Convoluted and deconvoluted native mass spectrums of the LHD29 heterodimer. **B:** Kinetic binding traces from BLI. Equilibrium responses were used to fit equilibrium binding curves **C:** Equilibrium binding curves of LHDs from biolayer interferometry binding assays with data from B. **D:** Equilibrium binding curves of  unfused LHD101 protomers binding to rigid DHR fusions of LHD101B (DHR4 and 62) and LHD101A (DHR21). Biotinylated unfused protomers were immobilized on streptavidin coated biosensors.


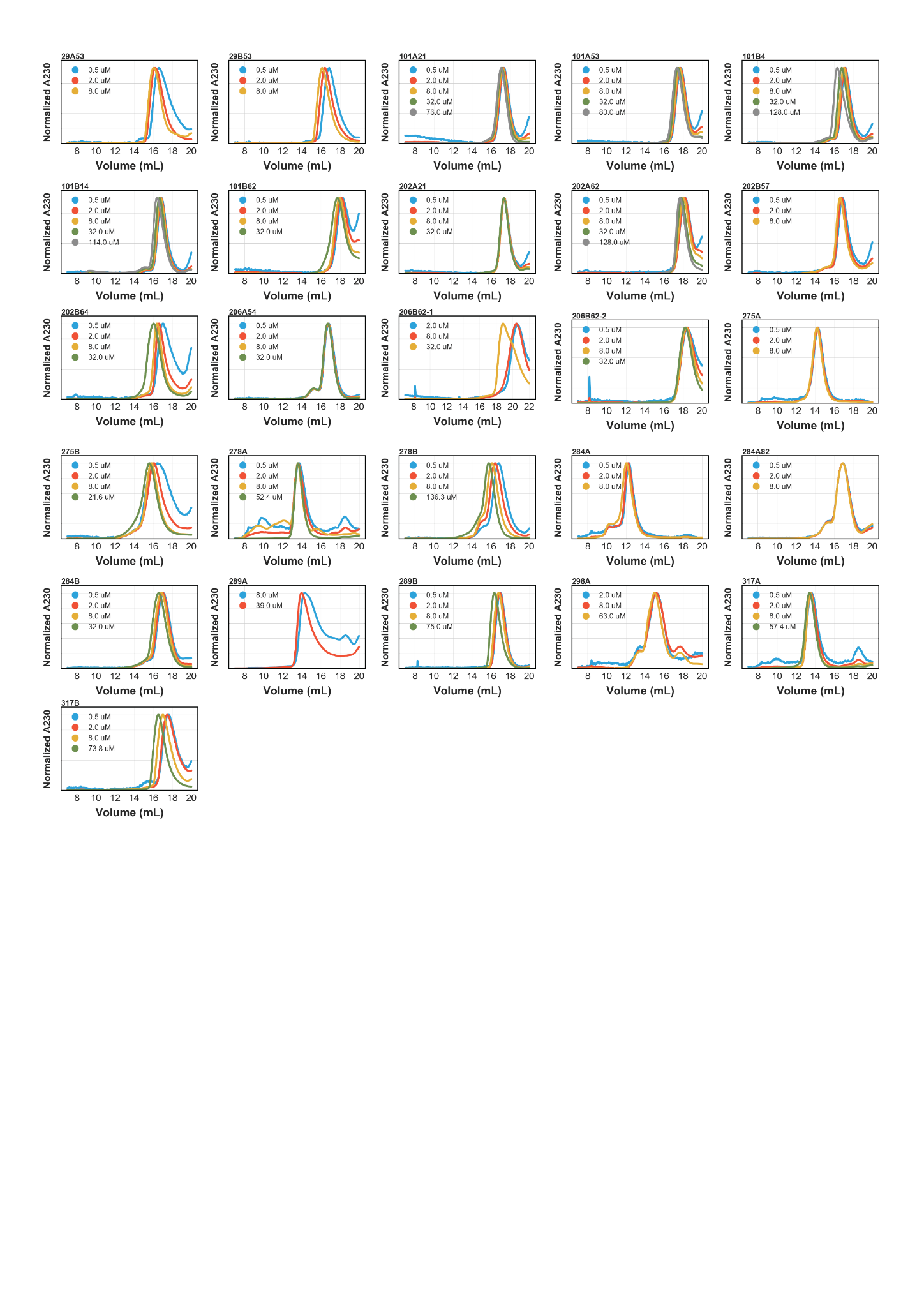
**Figure S4. Oligomeric state of LHD protomers.** SEC chromatograms of various LHD protomers titrated at indicated injection concentrations. All experiments were performed on a superdex 200 column except for LHDs 275A, 278A, 284A, 289A, 298A and 317A. These were run on a superdex 75 column. LHD protomers 29A53, 29B53, LHD202B and its variants, 275B, 278B and 289B tend to homodimerize at lower concentrations but still readily heterodimerize in presence of their designed partner.


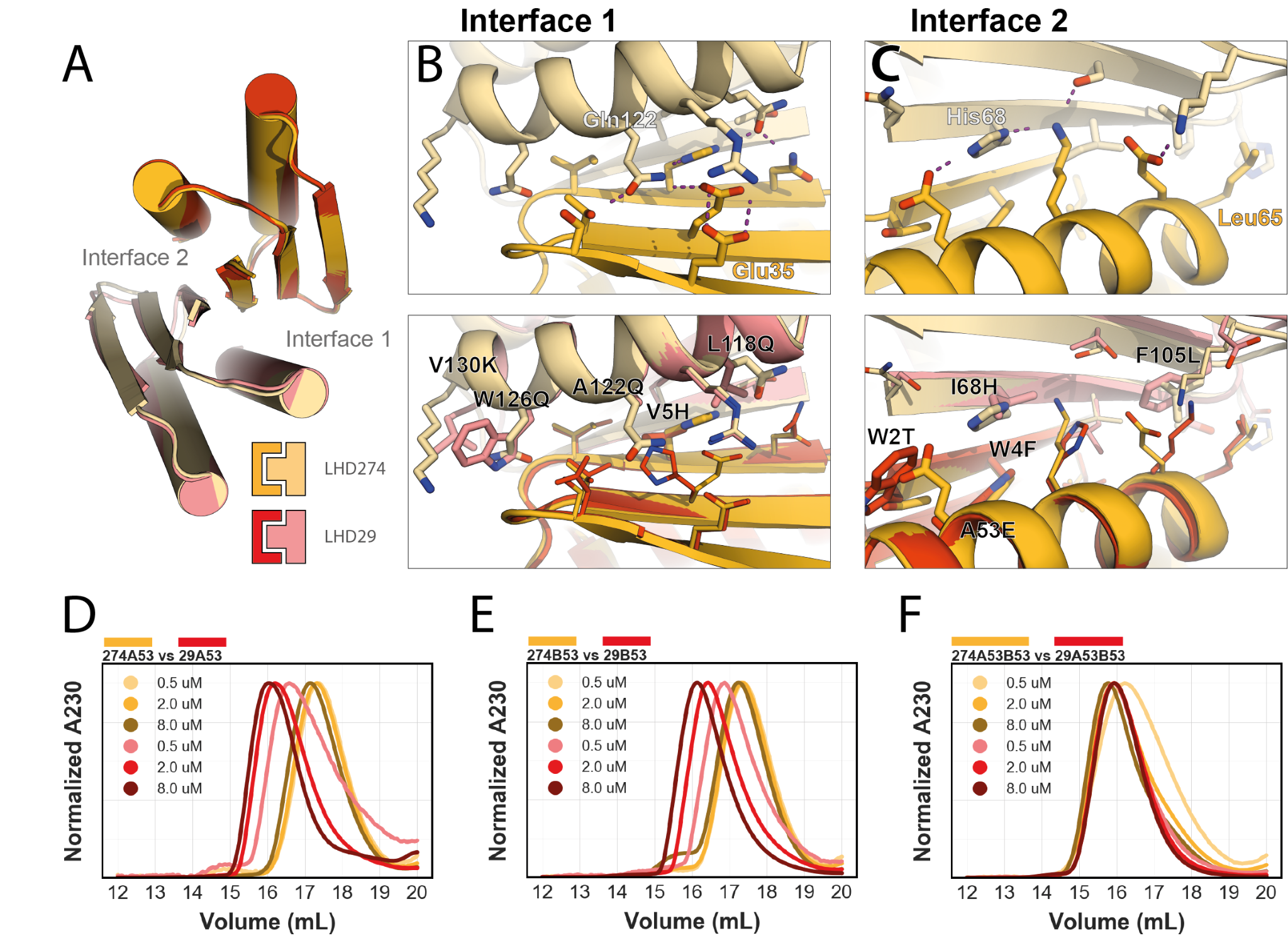


**Figure S5. Redesign of LHD29.** **A:** Superposition of a redesigned version of LHD29 designated LHD274 (yellows)  and LHD29 (reds). Top, atomic view of interface 1 (**B)** region of LHD29 and interface 2 region (**C)**. Bottom panels, Overlay view of LHD29 and LHD274 at the corresponding region. Thick sticks indicate hydrophobic to polar substitutions. **D:** SEC superdex 200 titration of LHD29A and LHD274A fused to DHR53 at indicated concentrations. Fusion proteins were chosen for this assay for their enhanced absorbance at 230 nm compared to the much smaller unfused versions. **E:** SEC superdex 200 titration of LHD29B and LHD274B fused to DHR53 at indicated concentrations. **F:** Titration of the 29 and 274 complexes.


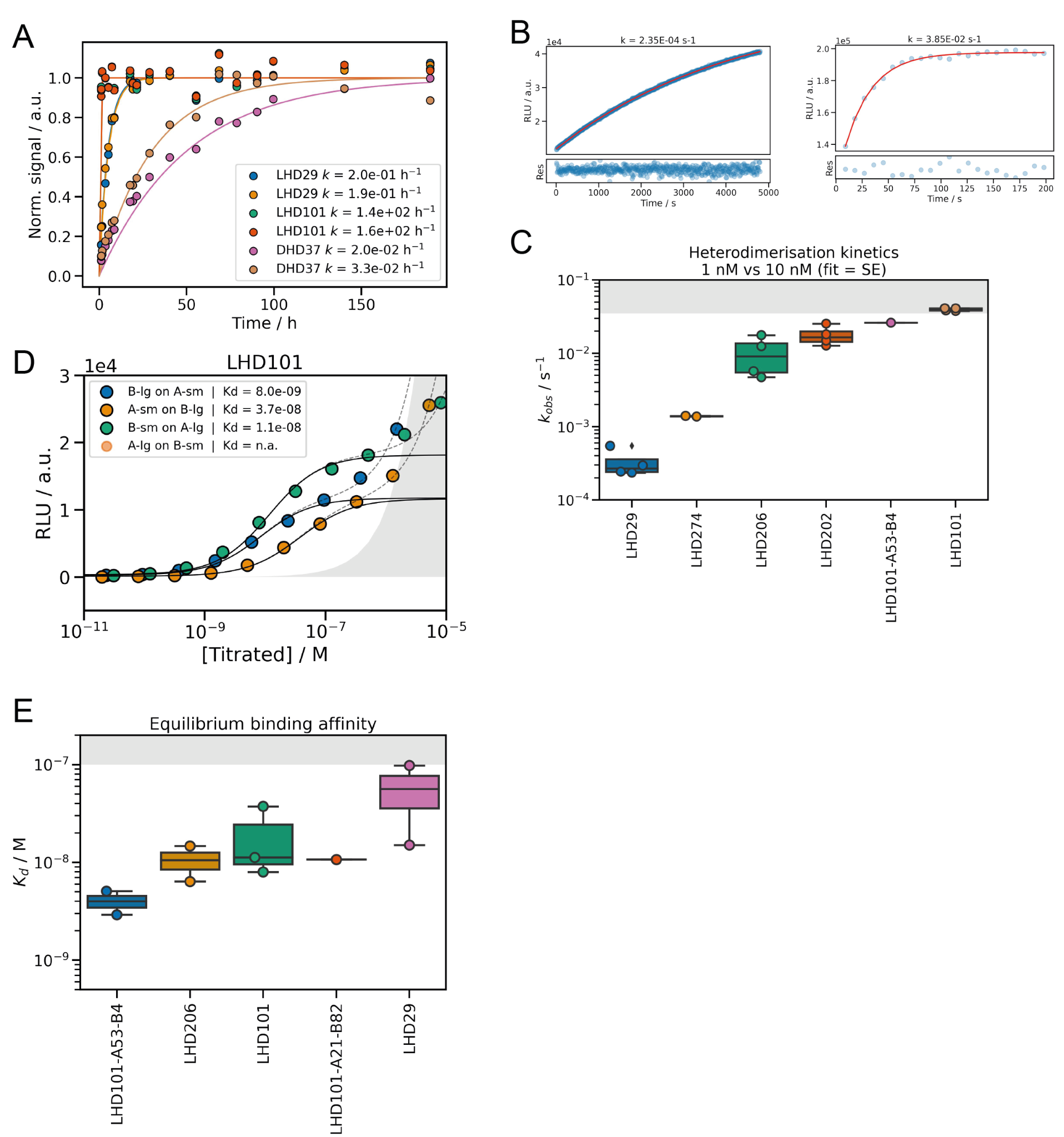


**Figure S6. Characterization of binding interactions with a split luciferase reporter assay.**

Protein interactions were characterized by monitoring the reconstitution of split luciferase activity (smBiT:lgBiT) upon binding in buffer (from purified components; A, G-H) or lysate (B-FE. **A** Comparison between the observed association kinetics of LHDs and designed helical hairpins (DHD37, previous work) under pseudo first-order conditions (1 nM *vs.* 10 nM). Reactions were monitored by taking manual time-points over the course of a week. The data was fitted to a single exponential decay function (solid line; rates are reported in the figure legend). **B** Example kinetic traces for the association of LHD29 (left) and LHD101 (right) in lysate. The data is shown in blue, and the single-exponential fits in red. Residuals to the fits are shown under each plot, and the rates are reported on top of each plot. **C** Summary statistics for association reactions performed under pseudo first-order conditions (1 nM *vs.* 10 nM) in lysate. Values are reported in Table S2. The grey shaded area indicates the limit of detection of the assay. **D** Example of equilibrium binding data collected in lysate (shown here for LHD101). Dashed lines are fits to the data, which includes a correction term to account for the intrinsic affinity of the split luciferase components (approximated by the grey shaded area). The binding curves (excluding the correction) are shown as solid black lines. The fitted *K*_d_ values are indicated in the figure legend. **E** Summary statistics for the equilibrium binding experiments performed in lysate. Values are reported in Table S3.


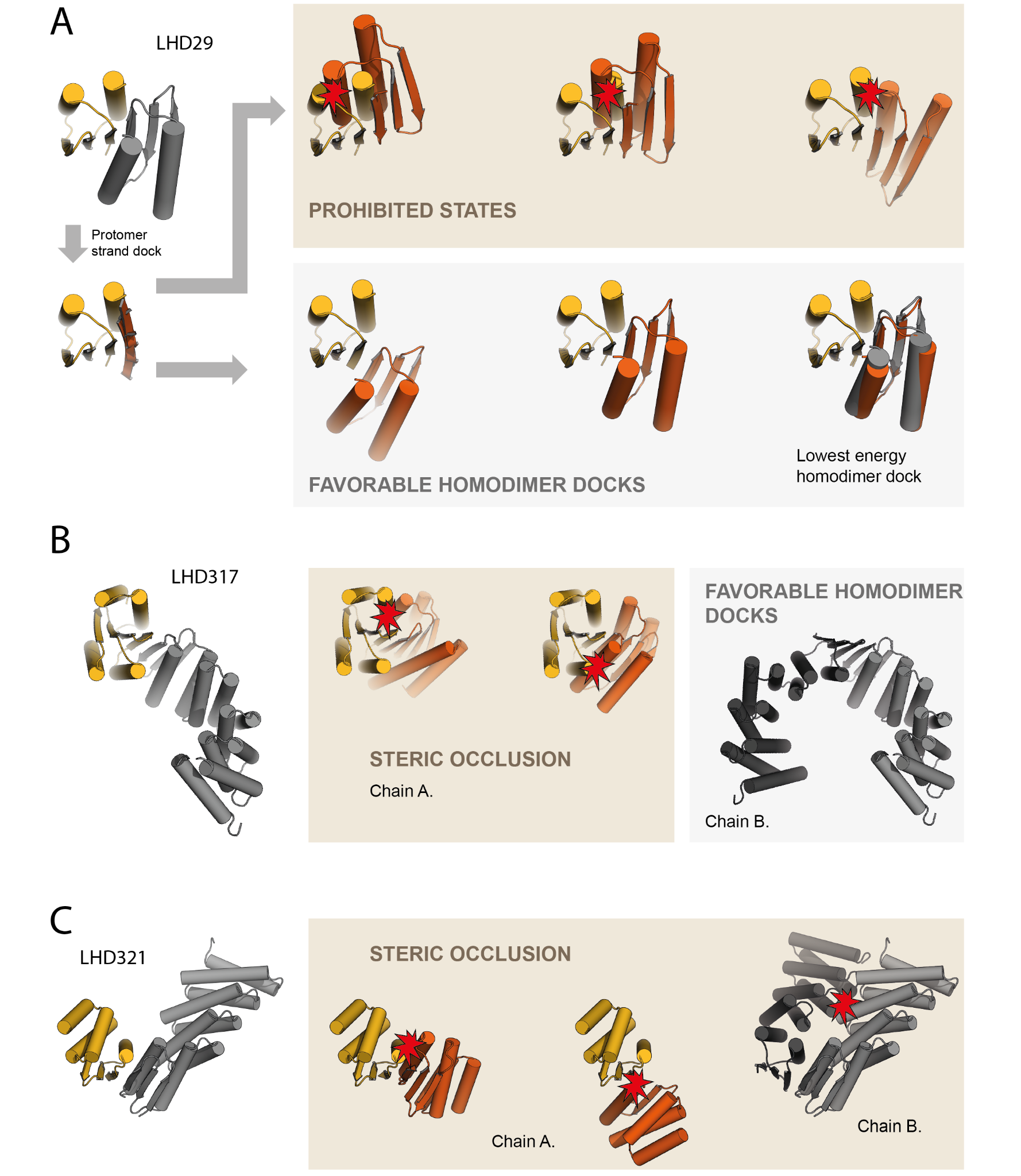


**Figure S7. Homodimer docking. A:** Example of homodimer docking. Homodimeric interaction most likely will occur on the edgestrand that forms the heterodimer. Strands are docked (orange) to the interface edgestrand of a protomer (yellow) of a given heterodimer. Another copy of the same protomer (orange) is then aligned along the docked edgestrand to create a homodimeric docked complex. Most complexes clash indicating homodimerization is unfavorable (top row). Some docks do not clash (bottom row) but have limited interaction surface area making homodimerization unlikely. In some cases homodimer docks i.e. LHD29 have similar interactions energies as the heterodimer (bottom right). These docks are likely to form homodimers. **B:** Homodimer docking of LHD317 protomers shows that secondary structure elements prevent LHD317A homodimerization via steric occlusion whereas 317B homodimers are more favorable. **C:** Designed secondary structure elements in both protomors of LHD321 prevent homodimerization


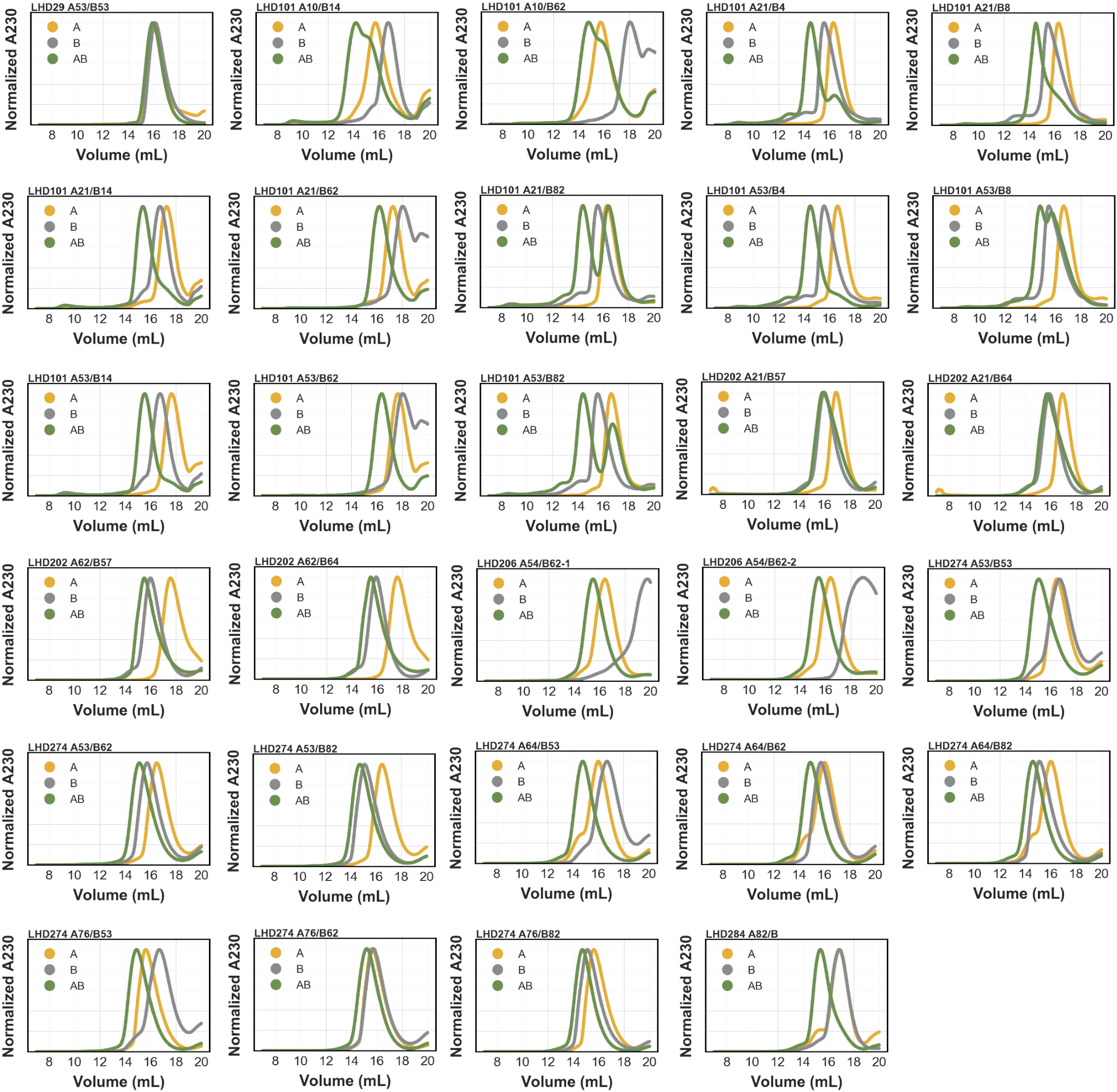


**Figure S8. LHD fusion binding assays.** Superdex 200 binding assays of LHD fusion proteins.


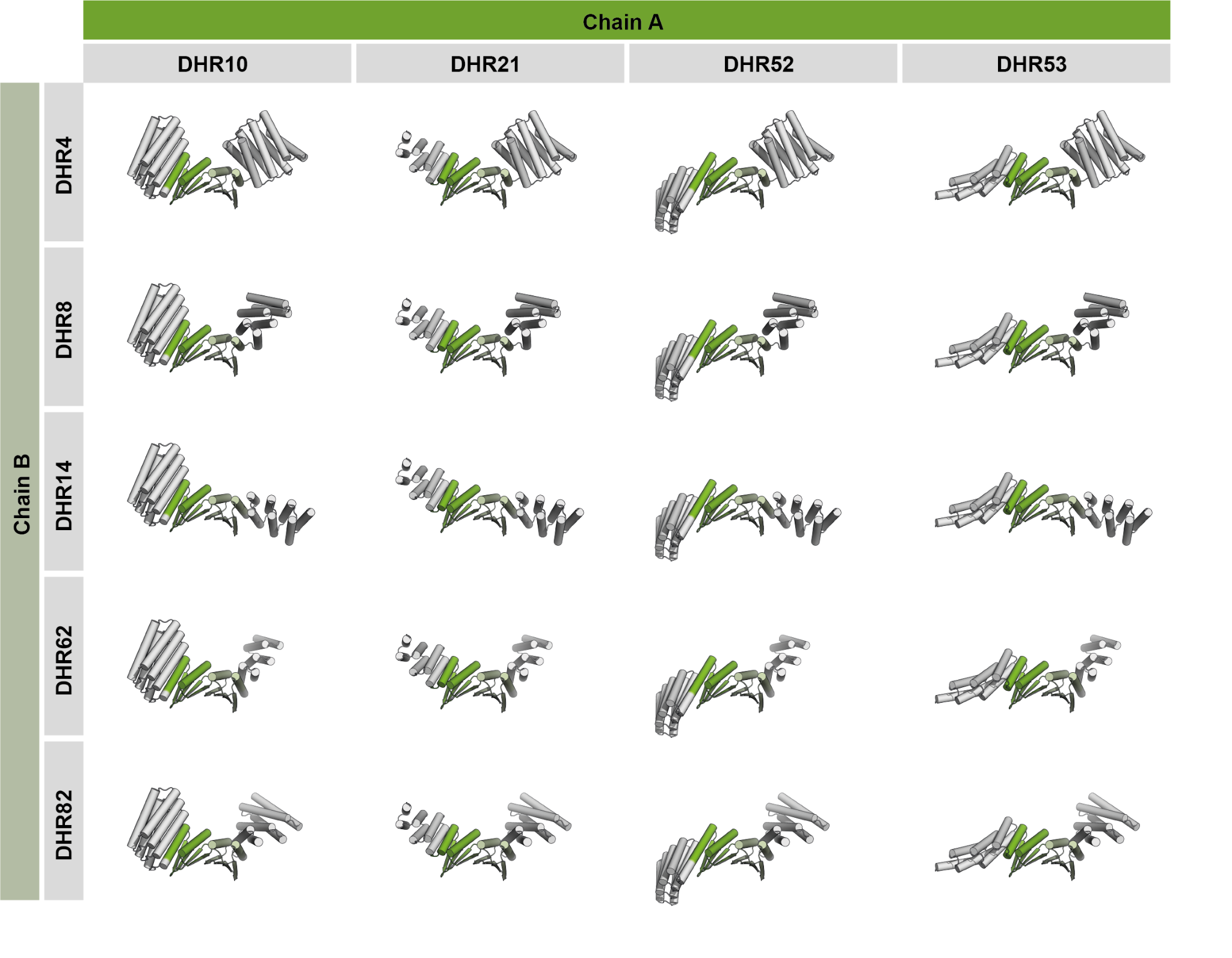


**Figure S9. Models LHD101 fusion complexes**. Designed models of all possible 20 complexes involving LHD101 fusions. DHRs colored in white and LHD base core protomers in greens. Combinations with unfused protomers (10 complexes) are not shown.


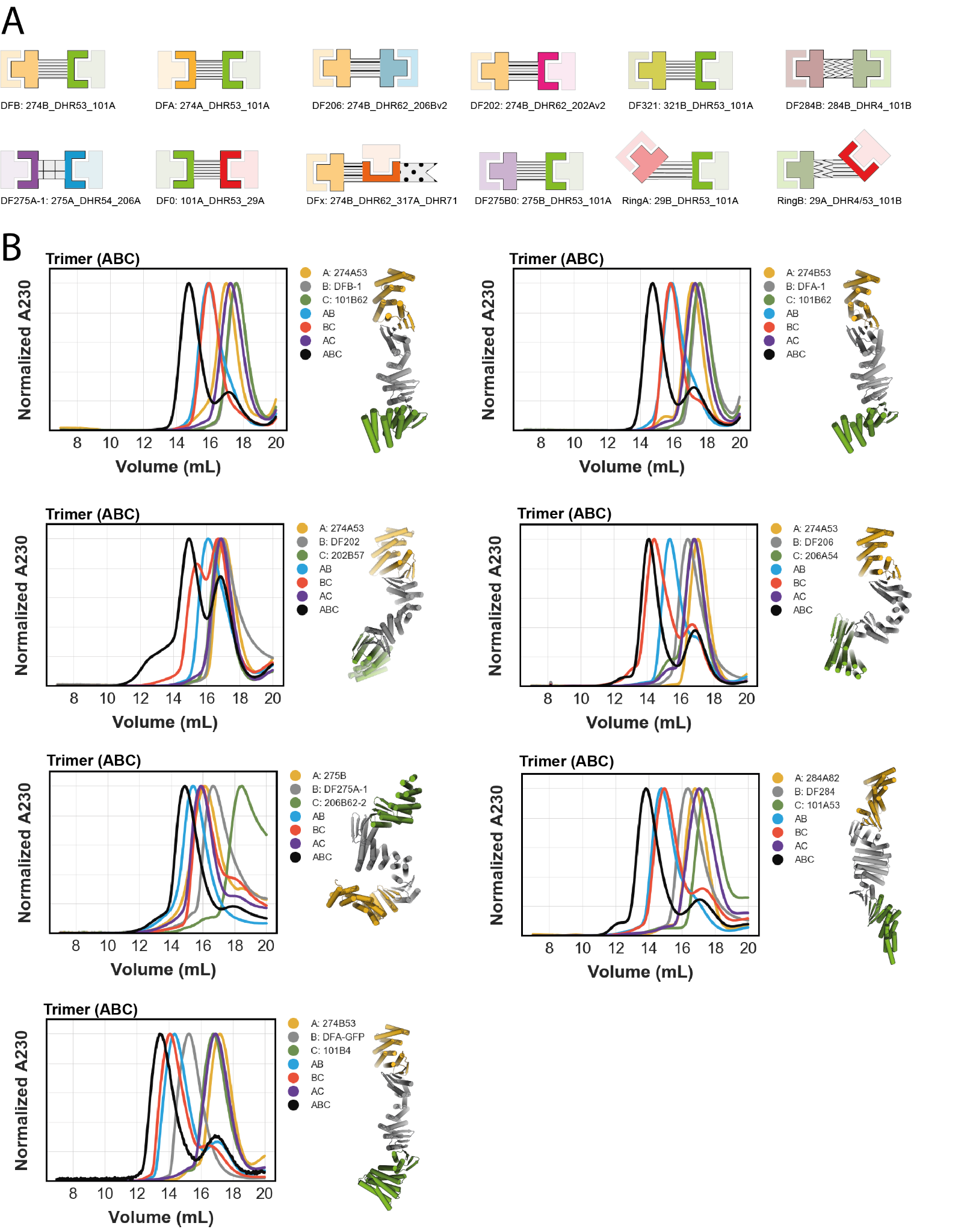


**Figure S10. Bivalent connectors and SEC binding assays linear heterotrimers. A:** Schematic overview of all bivalent connectors tested (see also supplementary excel file ). **B:** Superdex 200 chromatograms of various linear heterotrimers and their control sub-assemblies. Designed models of the target assembly (black chromatogram) are shown right of the graphs.


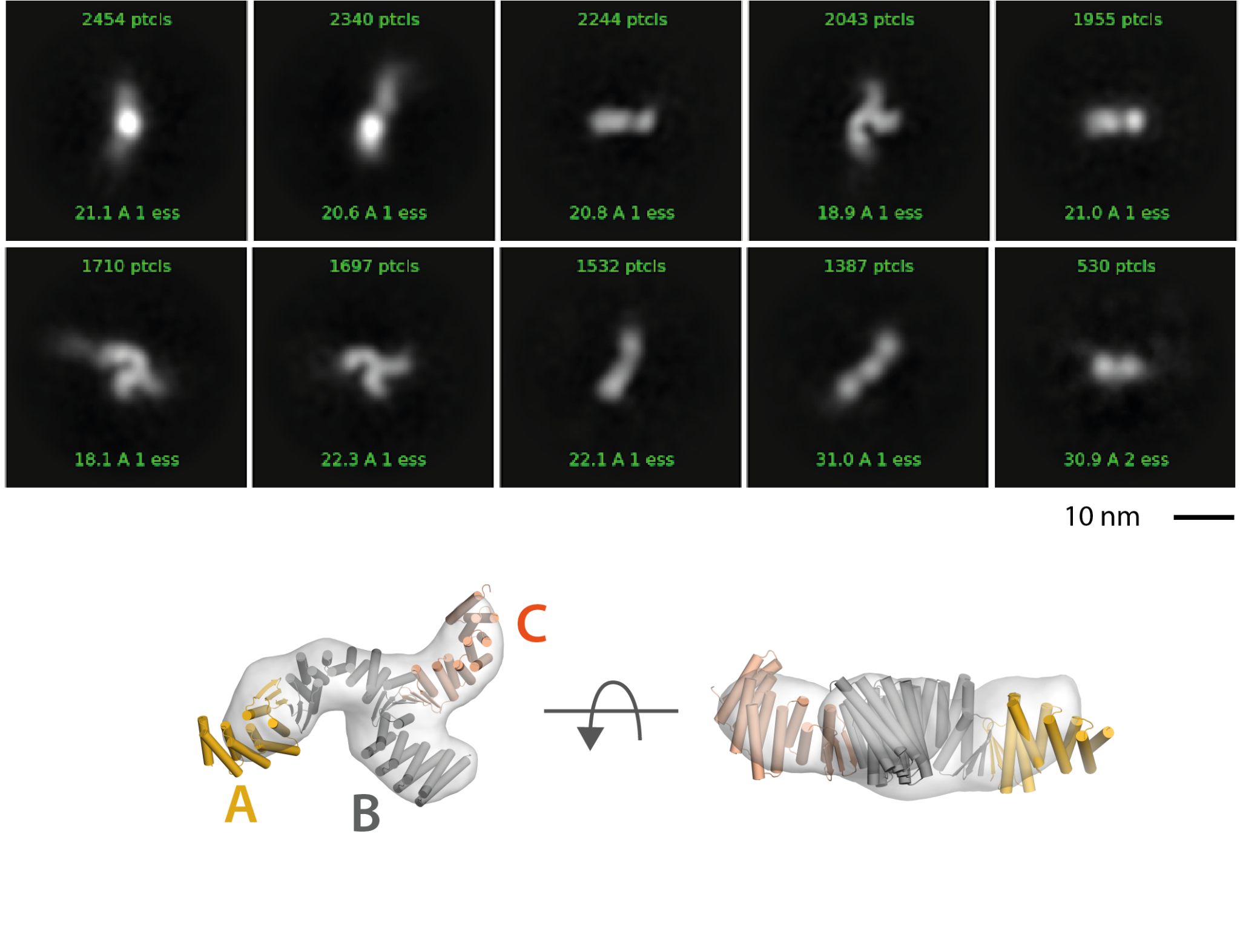


**Figure S11. Negative stain EM class averages and 3D reconstructions of a heterotrimer.** Heterotrimer (ABC) consisting of LHD274A53 (A), linear connector DFx (B) and LHD317B (C).


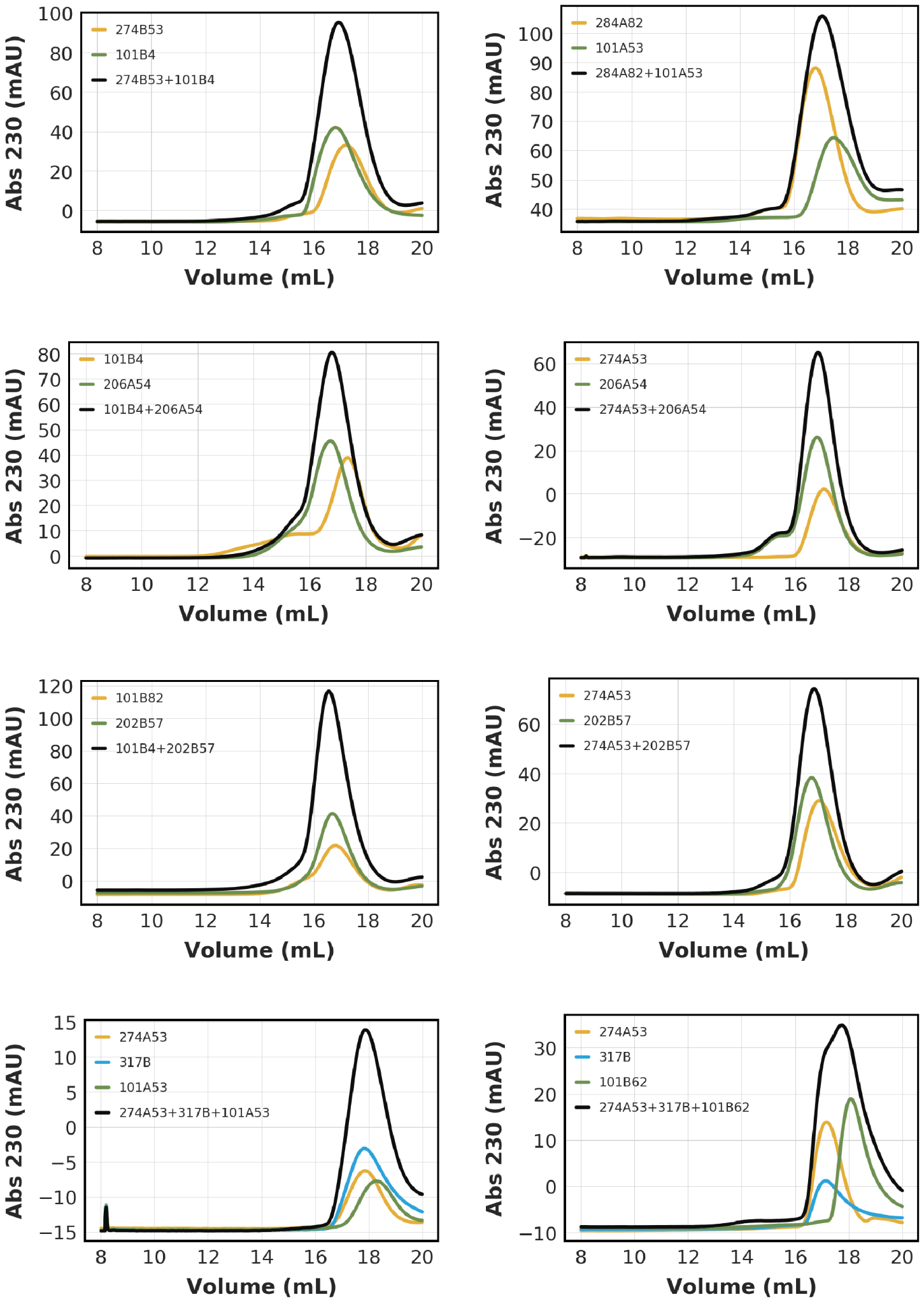


**Figure S12. Orthogonality SEC binding assays.** Superdex 200 chromatograms of eight samples that should not interact with each other indicating subunits presented in this work are specific.


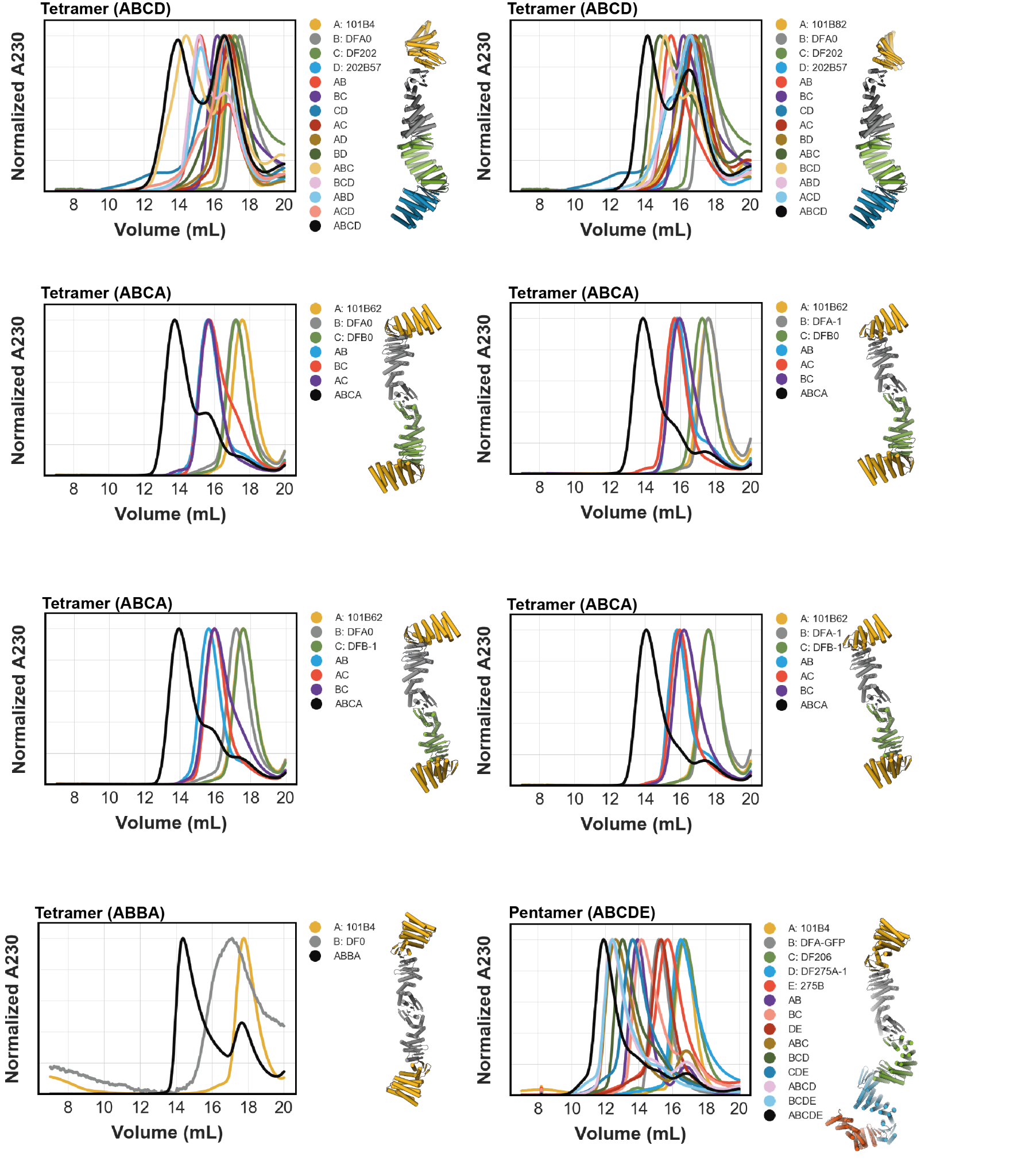


**Figure S13. SEC binding assays of linear heterotetramers and a heteropentamer.** Superdex 200 chromatograms of various linear heterotetramers and a heteropentamer, as well as their control sub-assemblies. Designed models of the target assembly (black chromatogram) are shown on the right of the graphs.


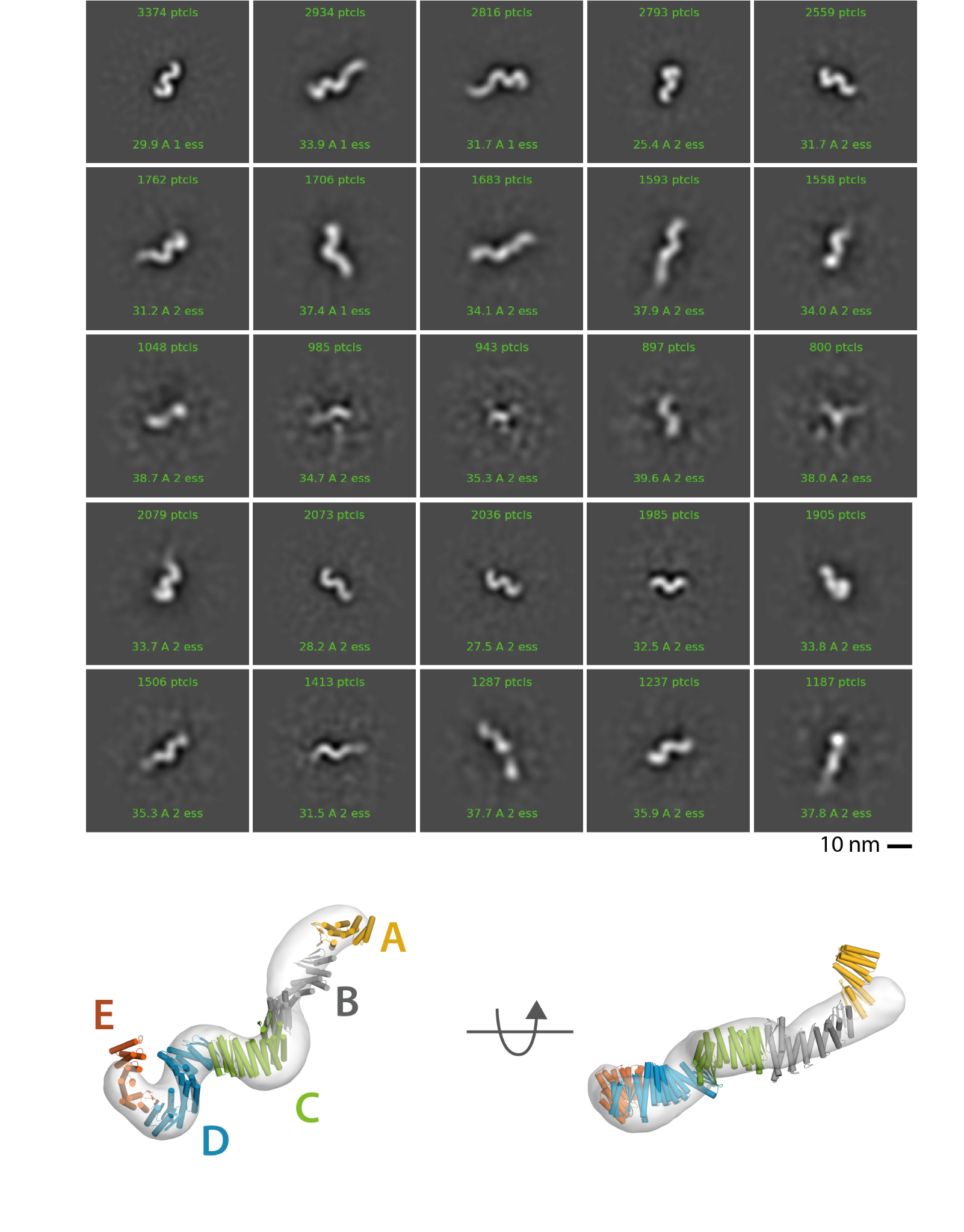


**Figure S14. Negative stain EM class averages and 3D reconstructions of a heteropentamer.** Heteropentamer (ABCDE) consisting of 101B4 (A), DFA0 (B), DF206 (C) , DF275A-1 (D) and 275B (E).


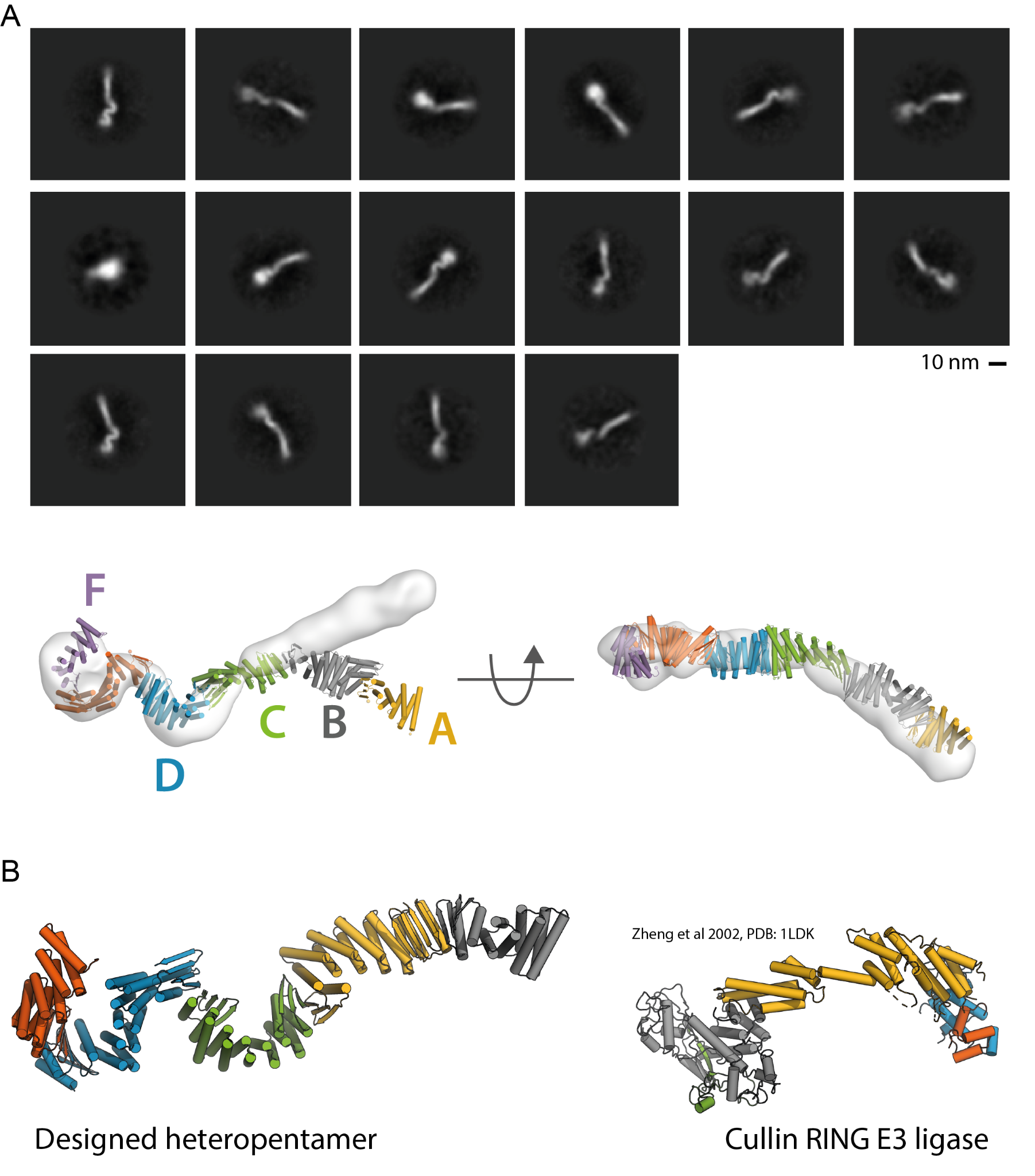


**Figure S15. Negative stain EM class averages and 3D reconstructions of the heterohexamer and comparison of designed assemblies to native complexes.** **A:** Heterohexamer consisting of 284A82 (A), DF284B (B), DFA0 (C), DF206 (D) , DF275A-1 (E) and 275B (F). **B:** Comparison between designed heteropentamer (left) and the Cul1-Rbx1-Skp1-F box^Skp2^ SCF ubiquitin ligase complex (right) [(Zheng et al. 2002)](https://paperpile.com/c/WUH5al/RK5v).


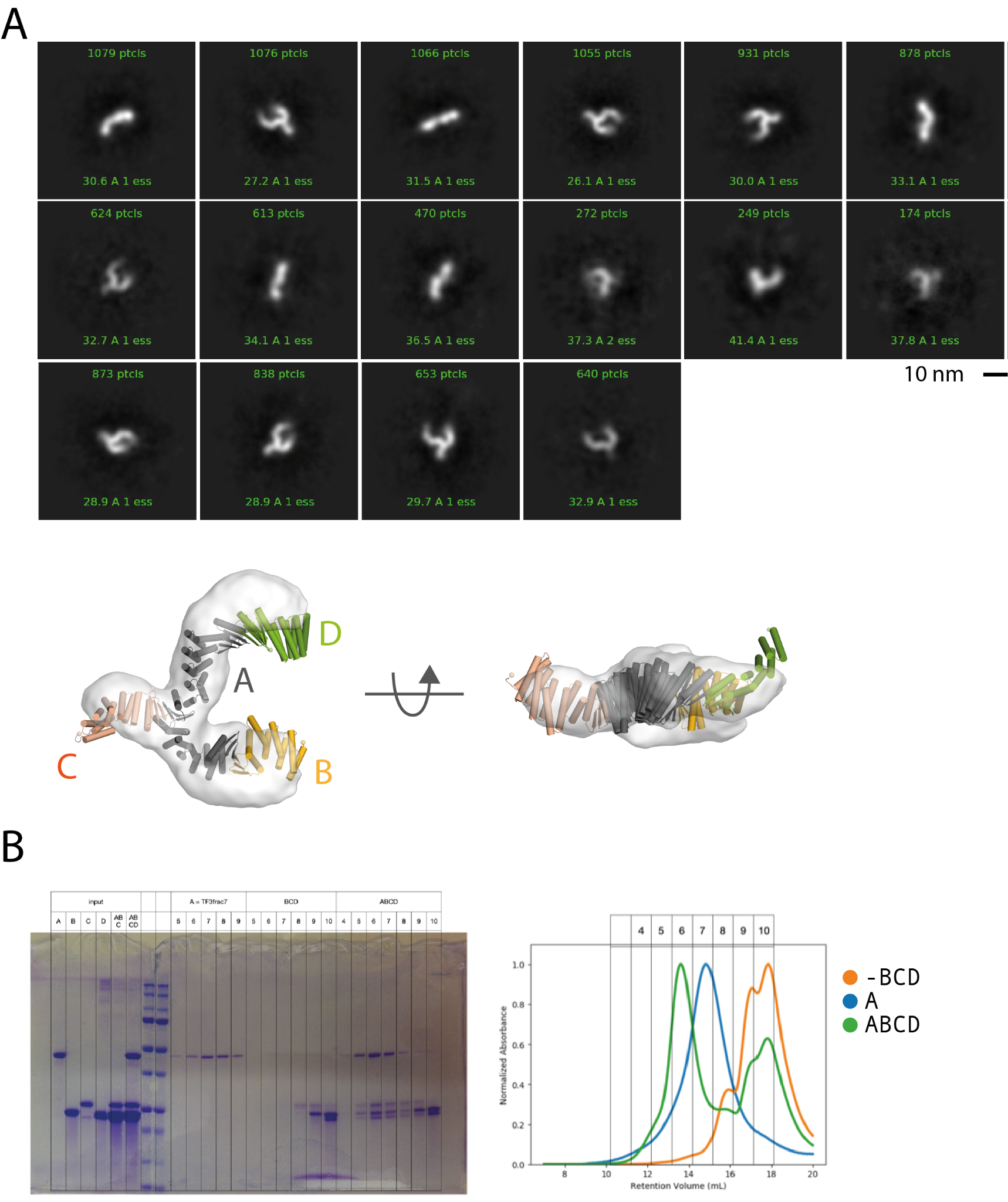


**Figure S16. Non-linearly branched assemblies. A:** Class averages and 3D reconstruction of a branched tetramer (ABCD) consisting of trivalent connector TF10 (A) and caps LHD274A53 (B), LHD317B (C) and LHD101B62 (D). **B:** SEC and corresponding SDS-PAGE analysis of a branched tetramer consisting of trivalent connector TF3 (A), LHD274A53 (B), LHD275B (C) and LHD101B62.


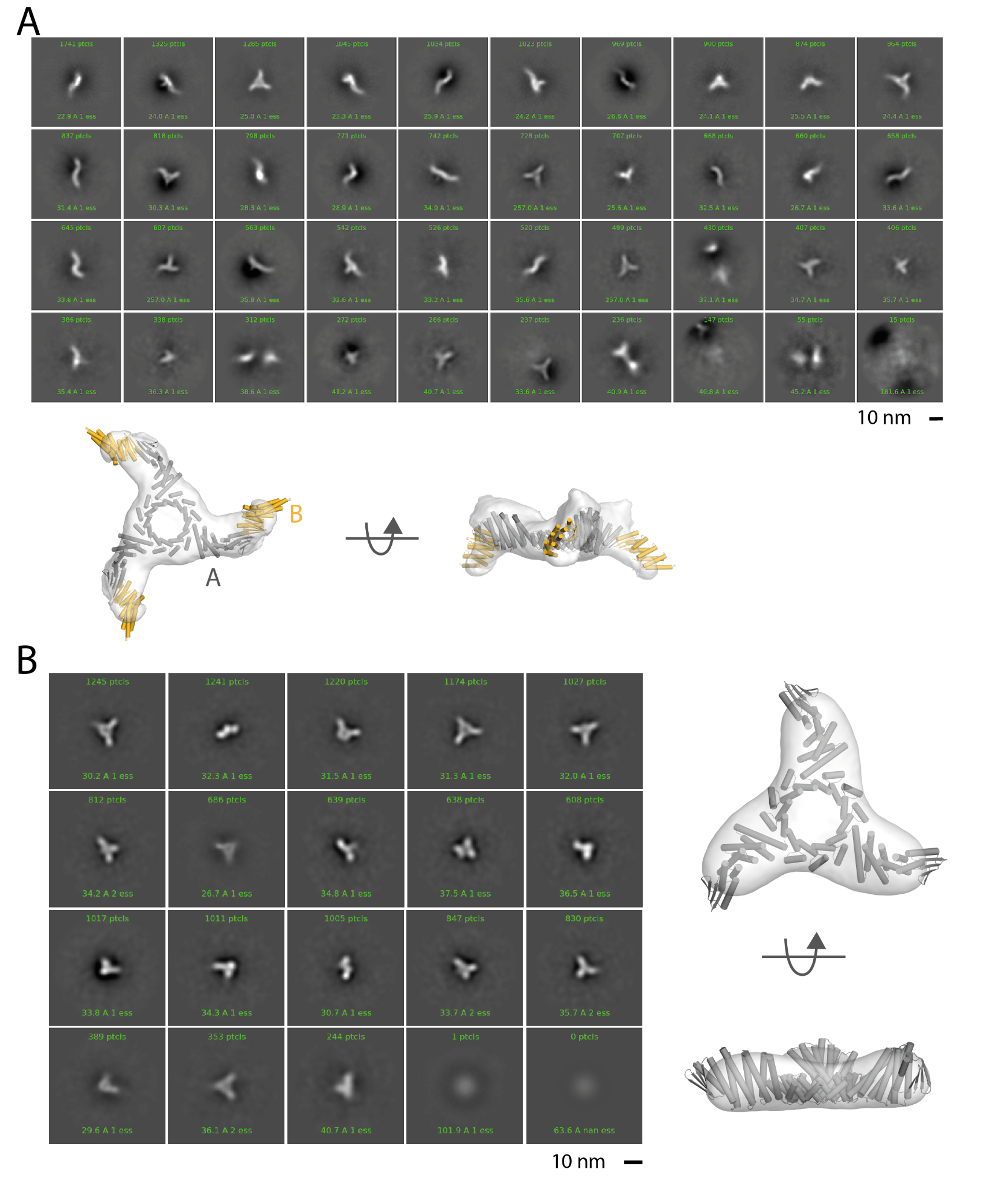


**Figure S17. Non linear assemblies on a C3 symmetric homo-oligomeric hub.** Class averages and 3D reconstruction  of the C3-Hub bound to LHD101A53 **(A)** and by itself **(B).**

**
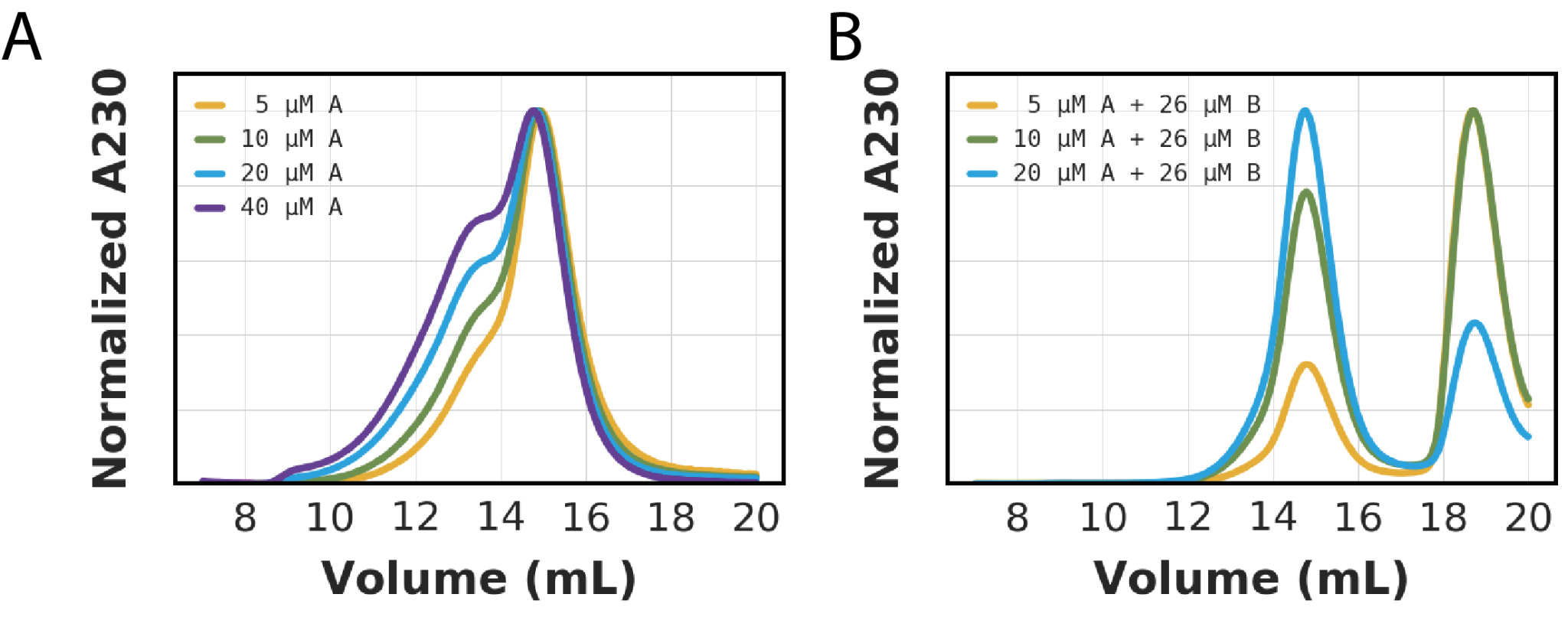
**

**Figure S18.**  **SEC Characterization of C4 symmetric hetero-oligomers.** SEC traces of the C4-symmetric hub at different concentrations without binding partner (**A)** and with a constant concentration of binding partner (**B**). Concentrations are given per monomer (5 µM corresponds to 1.25 µM tetramer).

**
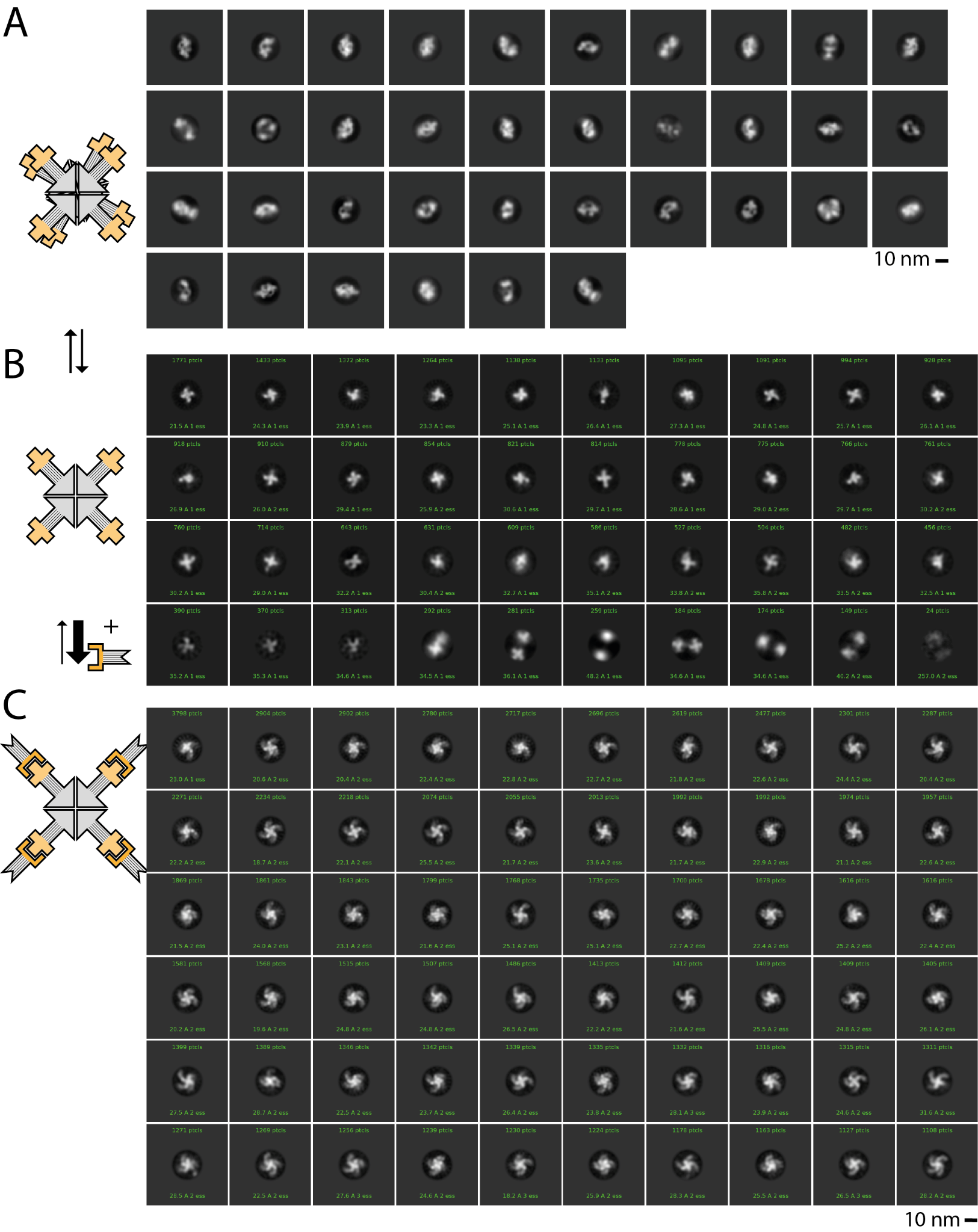
**

**Figure S19. Negative stain EM class averages of C4 symmetric hetero-oligomers.** Schematic representations (left; (dark grey: C4 hub, gold: binding partner) and negative stain EM class averages (right) of the  C4-symmetric hub without (**A**) and with (**C**) binding partner. In absence of the binding partner, the C4 hub exists in equilibrium between a higher order complex (**A**) and the designed C4 complex (**B**).


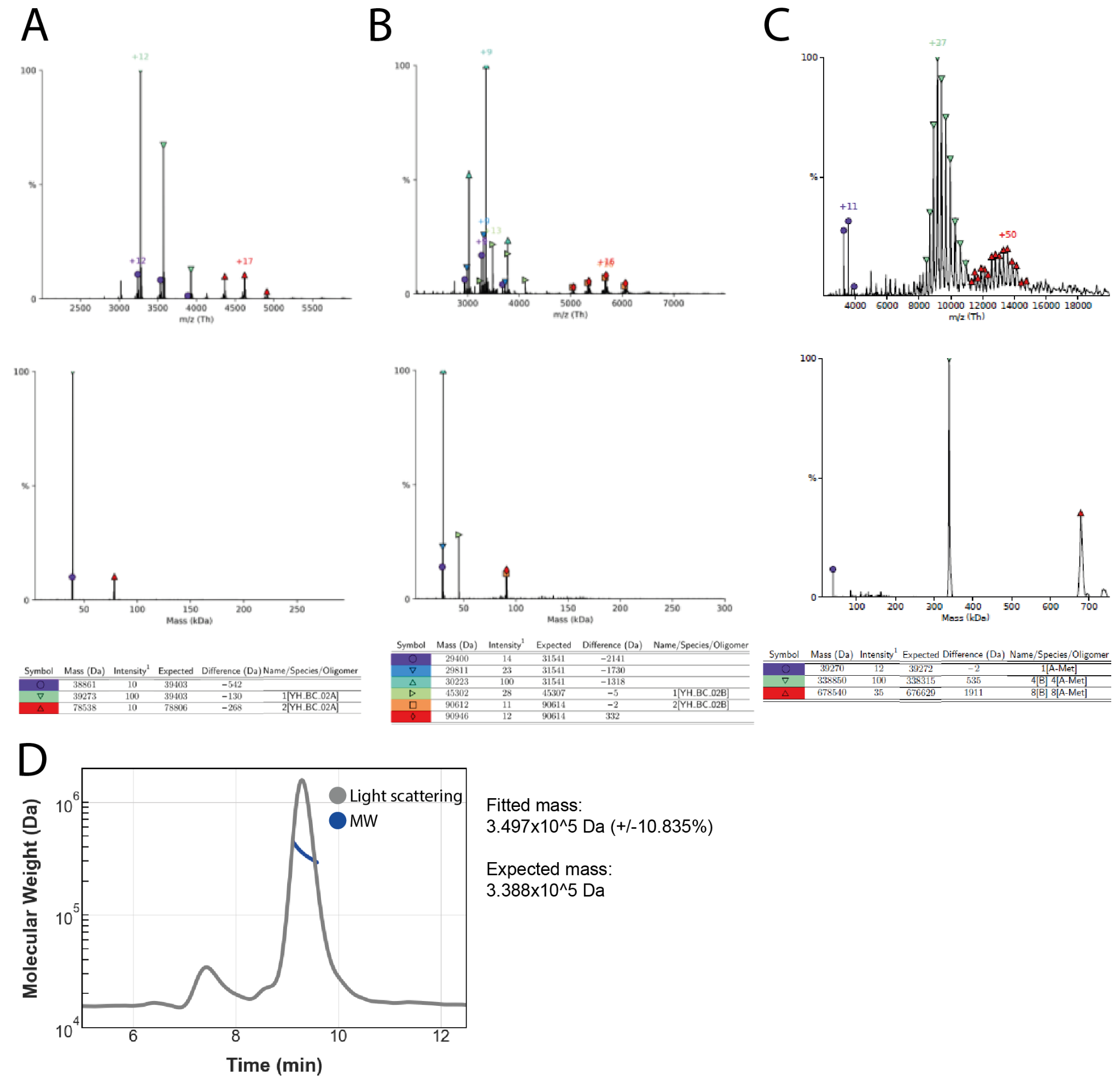


**Figure S20. Native mass spectrometry characterization of the two component closed C4-symmetric ring shown in Fig. 4D.** Convoluted (top) and deconvoluted (bottom) native mass spectrums of the constituent components (**A and B)** of two component C4-symmetrical and the ring assembly (**C).** **D:** SEC-MALS analyses of the ring with fitted peak mass and expected mass of the A4B4 complex.


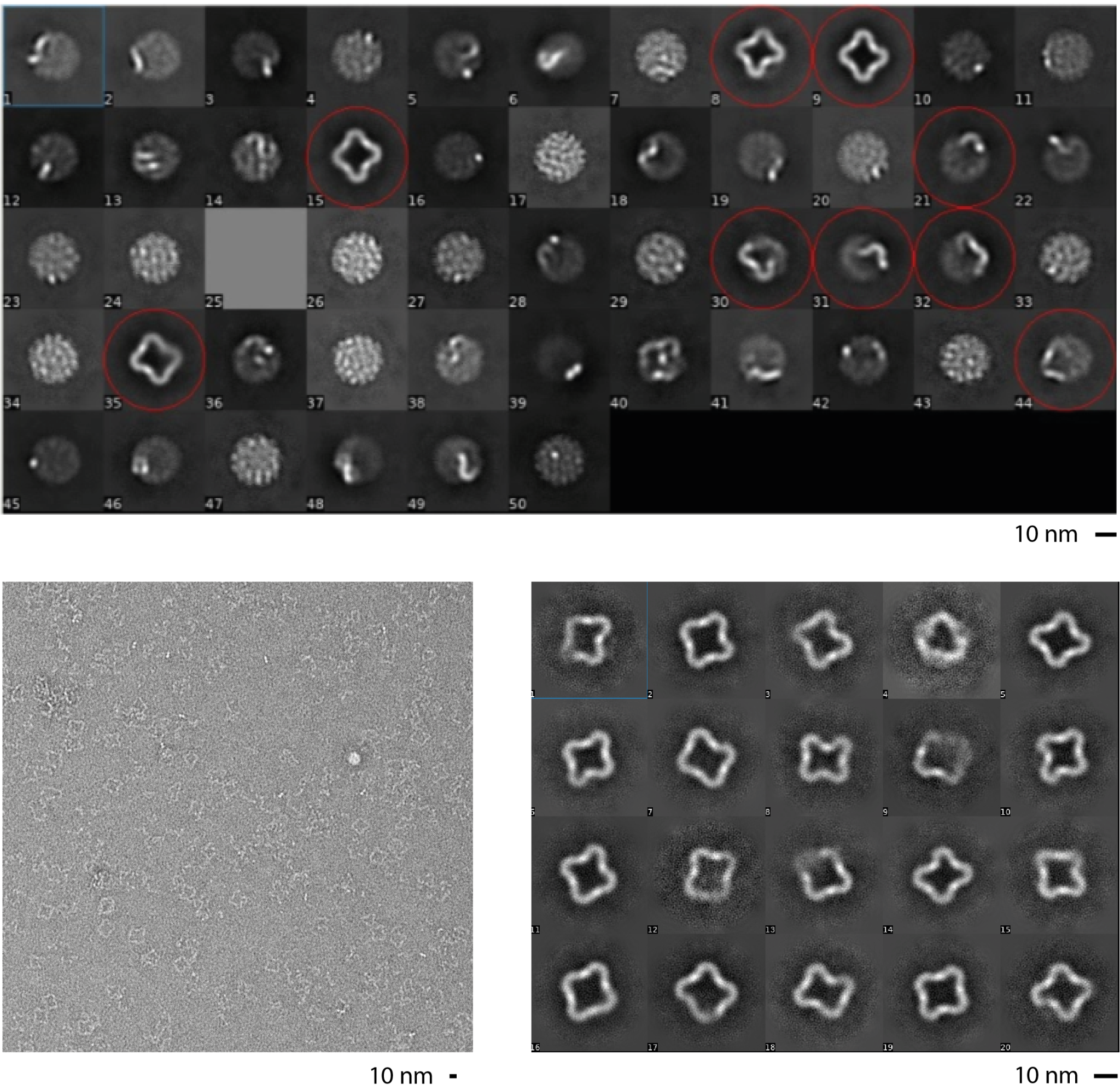


**Figure S21. Characterization of the two component closed C4-symmetric ring shown in Fig. 4D.** Top: Initial classification of nsEM particles. Red circles indicate classes that were used for a second round of classification. Bottom left: Raw negative stain electron micrograph of the ring. Bottom right: nsEM class averages of the closed C4-symmetric ring.


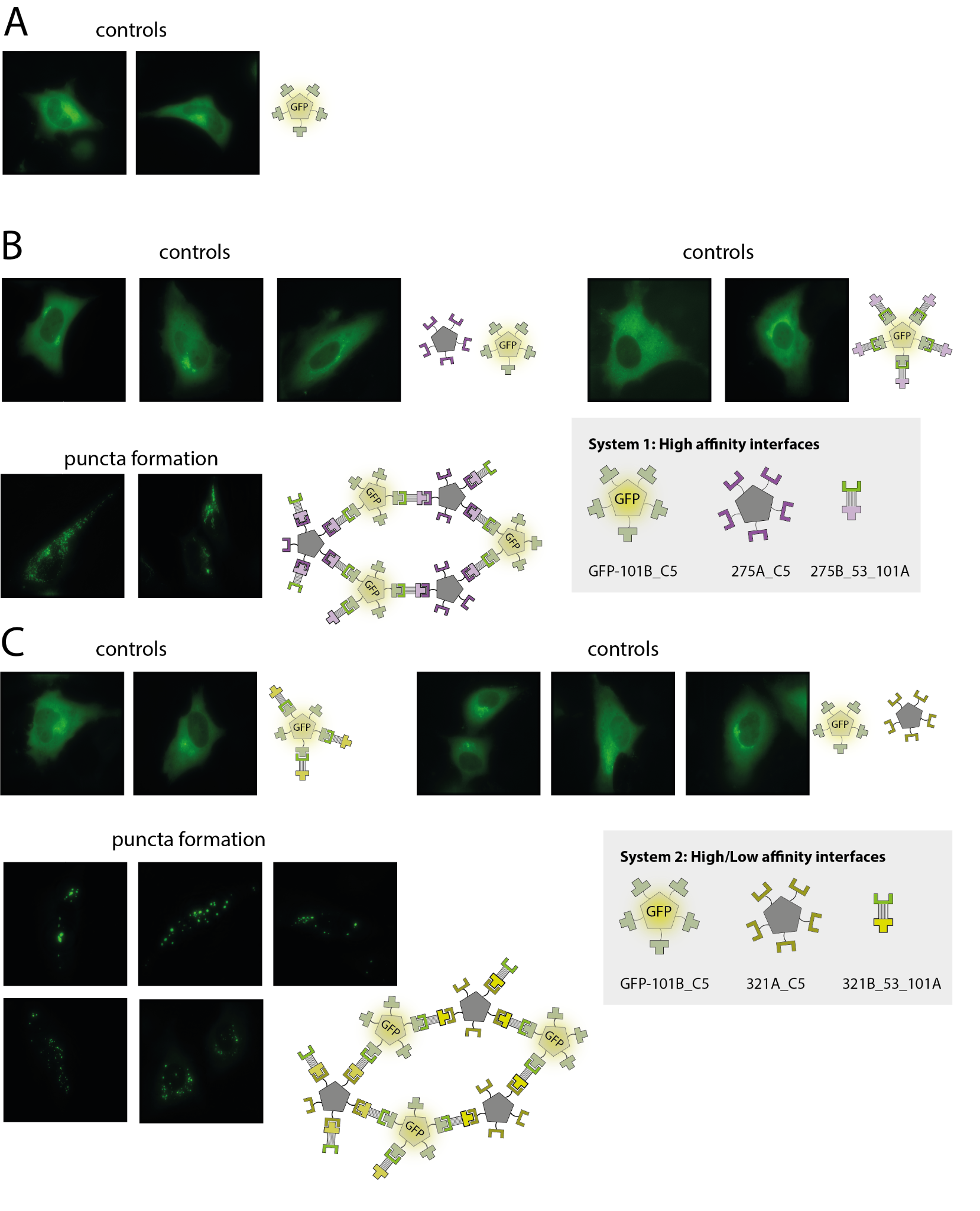


**Figure S22. Conditional binding in mammalian cells using 3 component assemblies.** Additional fluorescence microscopy images of the GFP channel of the control GFPtagged C5_LHD101 **(A)**, the high affinity system 1 using LHD101 and LHD275 interfaces **(B)** and the mixed high/low affinity system 2 **(C)** using LHD101 and LHD321 interfaces. Both LHD101 and LHD275 interfaces have low nanomolar Kd whereas the LHD321 interface Kd is low (likely micromolar, Low Kd made Kd determination challenging). Puncta formed in system 1 were typically small and numerous while punctas formed in system 2 were less numerous but larger and droplet-like. Fluorescent microscopy images are at the same scale as in Fig. 5A.


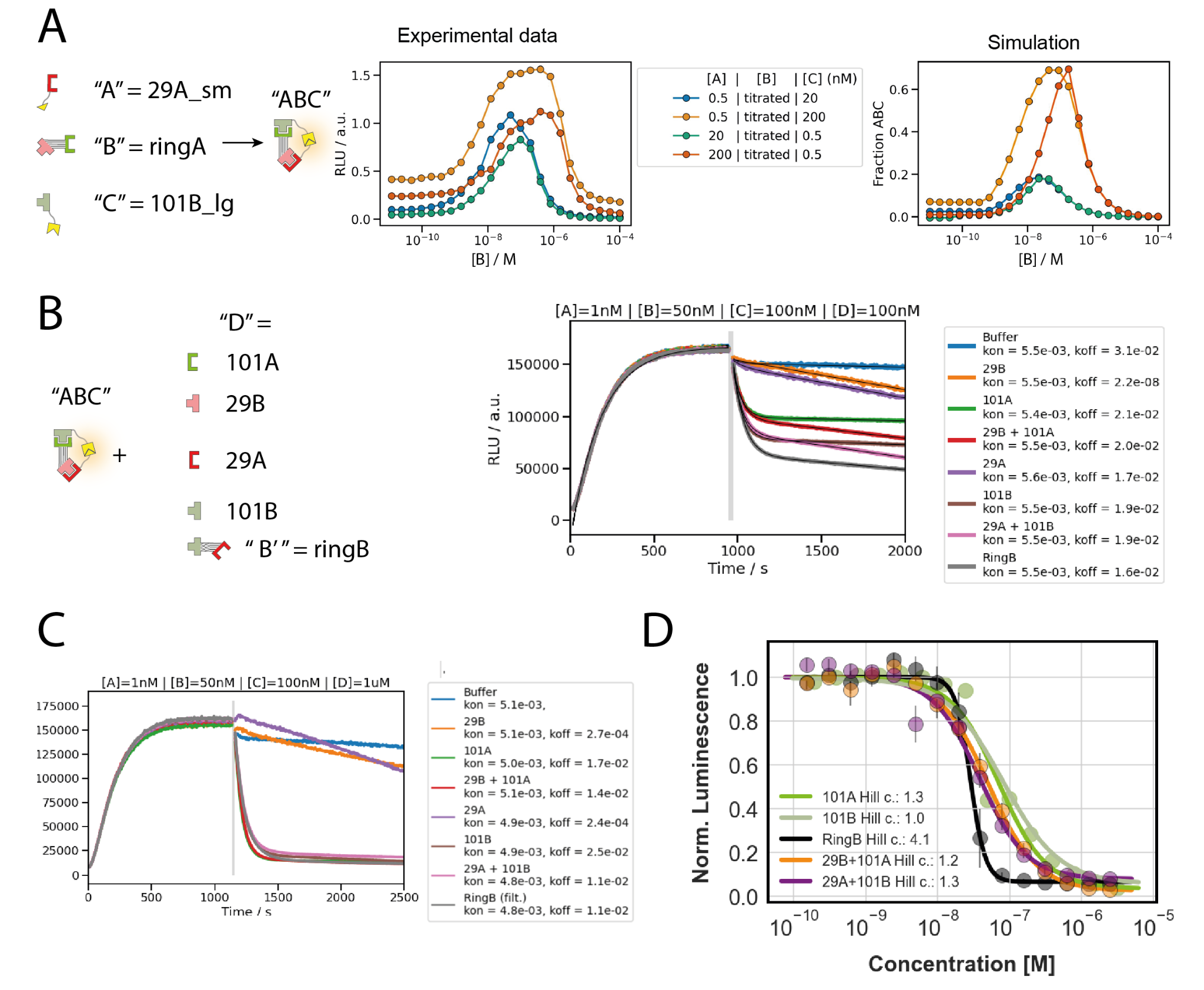


**Figure S23. Reconfiguration of protein assemblies through subunit exchange**. **A:** Formation of an ABC heterotrimer with split luciferase activity. Component A (LHD29A_sm) was tagged with the small part of the Nanobit luciferase and component B (LHD101B_lg) was fused to the large part of the Nanobit luciferase. Reconstitution of luciferase activity was followed by titration of component B (RingA, one of the components of the C4 two component ring). Left: schematic representations of the components; Center: Experimental determination of trimer formation at different concentrations of the components; Right: Simulated assembly yield of the trimer at different concentrations of the components, assuming KD_LHD29 = 50 nM and KD_LHD101 = 5 nM. The ABC heterotrimer with luciferase activity in Fig. 5 and Fig. S23B-D was assembled using 1 nM, 50 nM and 100 nM of components A, B and C respectively. **B:** Left: Schematic representation of the components for the reconfiguration assay; Right: Kinetic assay where an ABC heterotrimer is disassembled by the addition of 100 nM of different variations of component D (see legend) at the indicated time point (gray vertical bar). **C:** Same as **B.** but adding 1000 nM of component D. **D:** Titration of various designs to a preformed ABC heterotrimer. Only the titration of RingB leads to cooperative change in luminescence indicating ring formation by association of RingA to RingB. Data fitted to the hill equation. Error bars represent sd.


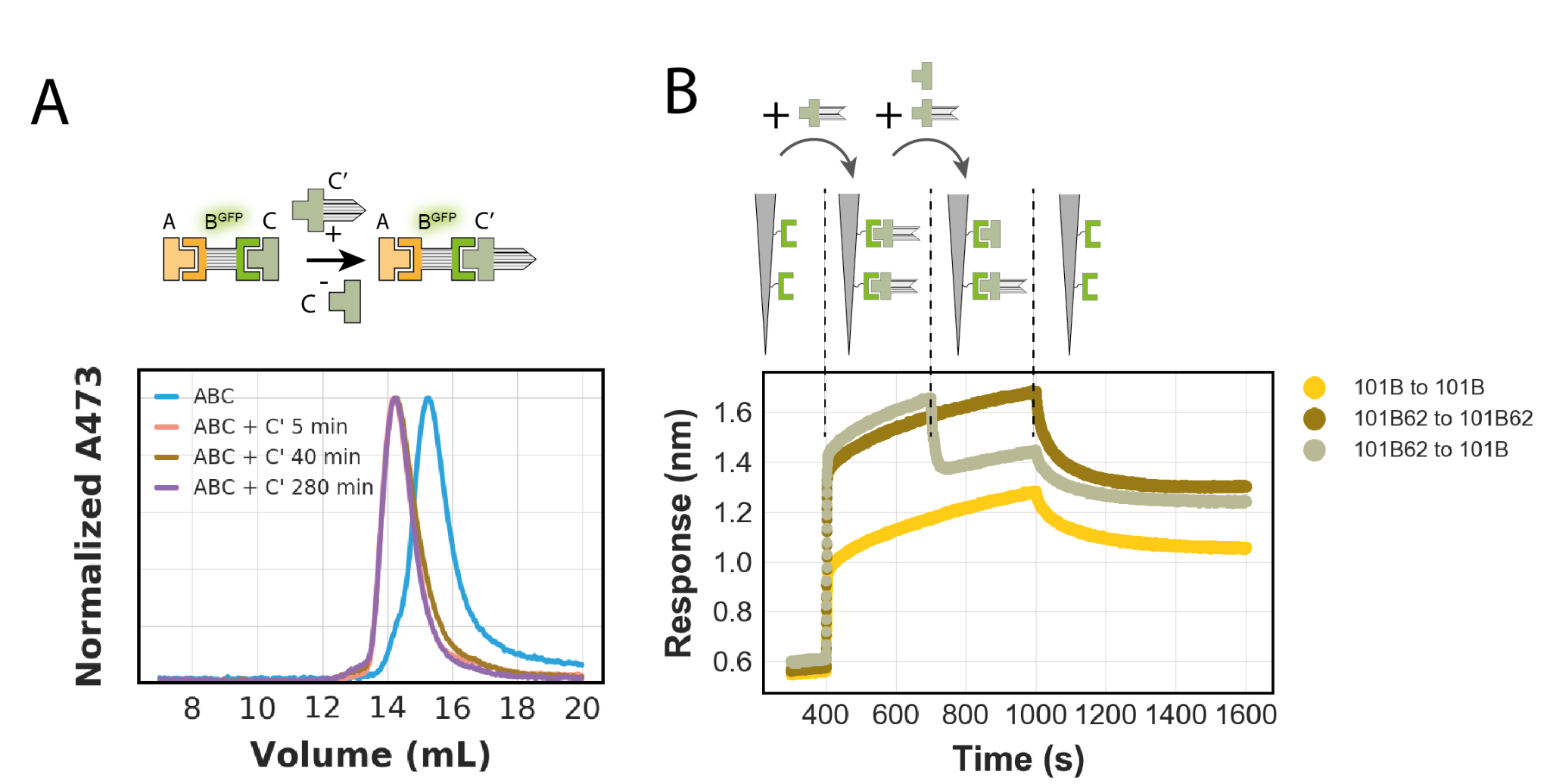


**Figure S24. Reconfiguration of protein assemblies through subunit exchange**. **A:** Exchange experiment in which a pre-assembled trimer (“ABC”, A=LHD274B, B=GFP-DFA0, C=LHD101B) is incubated with a variant of one of the components (“ C’  ”, C'=LHD101B62). Top: Schematic representation, bottom: SEC traces of trimer mixture before and after addition of component C’ indicating that C can be exchanged by C'. **B:** Biotinylated LHD101 that is immobilized to streptavidin biosensors binds rigid fusion variant LHD101B62. Biosensors were next dipped into a solution containing equimolar amounts of LHD101B62 and unfused 101B at saturating concentrations. The binding response of this reaction is in between controls (brown and yellow) indicating subunit exchange takes place.


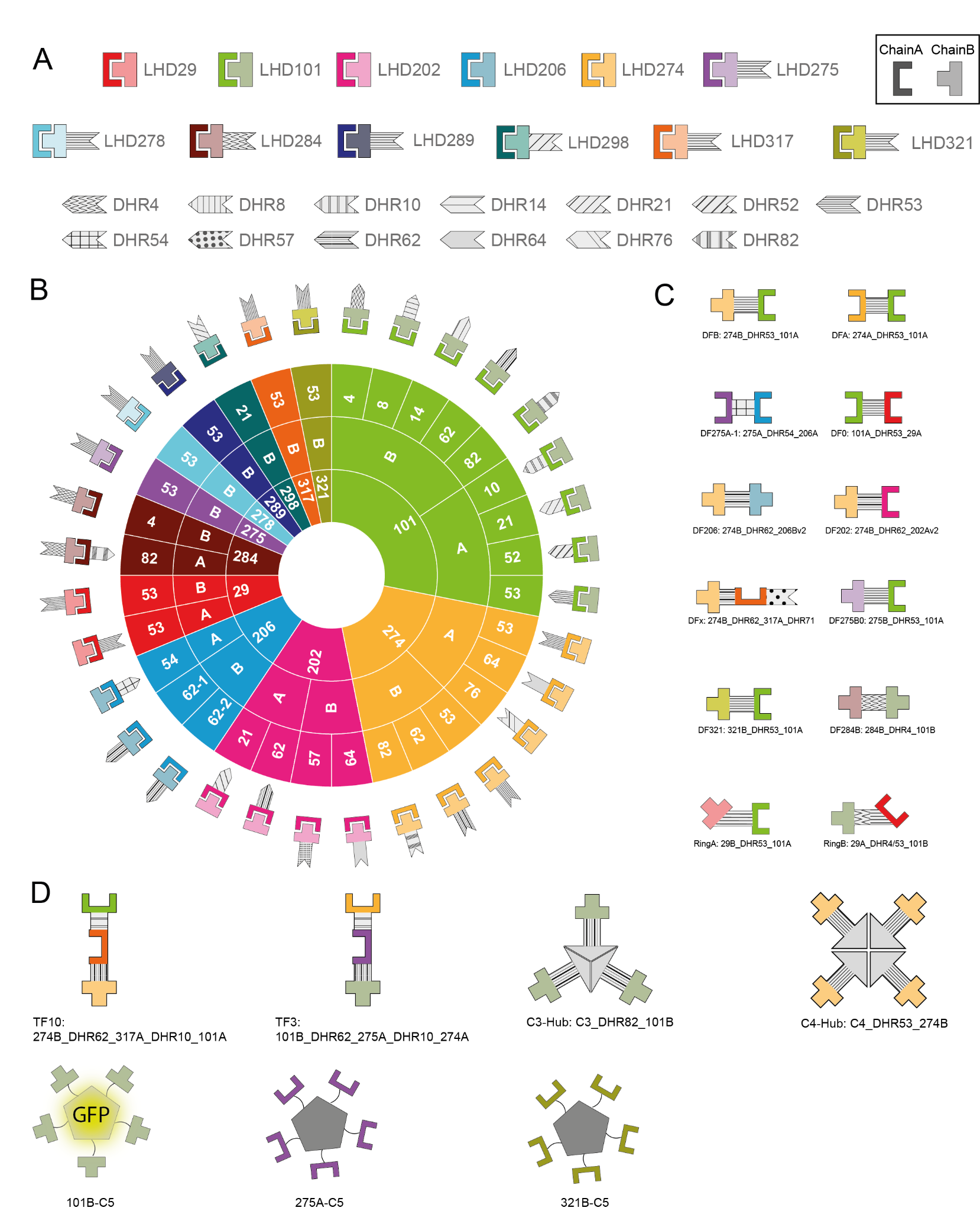


**Figure S25. Overview of all protein components used in this work. A:** Top two rows: Base heterodimers; bottom two rows: unfused DHRs. **B:** Sunburst plot of rigid fusions of heterodimers to DHRs. **C:** Bivalent connectors. **D:** Trivalent connectors and homo-oligomeric hubs. Color schemes for heterodimers and patterns for DHRs are used consistently through all figures. Names of individual components are consistent with the information in the supplementary excel file.

**Table S1.** Fitted values biolayer interferometry binding assays

| **Design** | **Steady state fits** | | **Kinetic fits** | | | | |
| --- | --- | --- | --- | --- | --- | --- | --- |
|  | **K_D_ (nM)** | **R-sqr** | **K_D_ (nM)** | ***k*_on_ (M^-1^ s^-1^)** | ***k*_off_ (s^-1^)** | **chi-sqr** | **R-sqr** |
| **LHD29^1^** | 310 ± 120 | 0.91 | 985 ± 6.0 | 6.9·10^2^ ± 4 | 6.8·10^-4^ ± 1.1·10^-6^ | 6.7 | 0.98 |
| **LHD101** | 9.5 ± 0.76 | 0.99 | 1.9 ± 0.04 | 2.2·10^6^ ± 4.0·10^4^ | 4.3·10^-3^ ± 2.1·10^-5^ | 0.21 | 0.97 |
| **LHD202** | 2400 ± 170 | 0.99 | 4800 ± 250 | 6.0·10^4^ ± 3.0·10^3^ | 2.9·10^-1^ ± 0.05 | 0.03 | 0.99 |
| **LHD206** | 8.4 ± 1.6 | 0.97 | 2.8 ± 0.02 | 2.7·10^5^ ± 1.9·10^3^ | 7.5·10^-4^ ± 2.2·10^-6^ | 0.8 | 0.99 |
| **LHD274** | nd | nd | nd | nd | nd | nd | nd |
| **LHD275** | 4.5 ± 0.22 | 0.99 | 2.9 ± 0.01 | 1.4·10^5^ ± 4.6·10^2^ | 4.1·10^-4^ ± 1.1·10^-6^ | 0.76 | 0.99 |
| **LHD278** | 3.4 ± 0.69 | 0.98 | 0.8 ± 0.003 | 2.9·10^5^ ± 1·10^3^ | 2.2·10^-4^ ± 3.6·10^-7^ | 2.8 | 0.99 |
| **LHD284** | 97 ± 13 | 0.99 | 8.9 ± 0.13 | 1.3·10^5^ ± 1.7·10^3^ | 1.2·10^-3^ ± 6.7·10^-6^ | 0.06 | 0.99 |
| **LHD289** | 610 ± 120 | 0.97 | 1080 ± 39 | 5.3·10^4^ ± 1.9·10^3^ | 5.7·10^-2^ ± 5.8·10^-4^ | 0.99 | 0.99 |
| **LHD298** | 16 ± 3 | 0.97 | 3.5 ± 0.01 | 6.4·10^4^ ± 1.0·10^2^ | 2.2·10^-4^ ± 5.9·10^-7^ | 6.4 | 0.99 |
| **LHD317** | 56 ± 2.3 | 0.99 | 34.7 ± 0.05 | 1.5·10^5^ ± 2.1·10^3^ | 5.1·10^-3^ ± 1.6·10^-5^ | 4.7 | 0.99 |
| **LHD321^2^** | nd | nd | nd | nd | nd | nd | nd |

^1^Homodimerization of both LHD29 protomers under BLI conditions make Kd determination unreliable. Kd from split luciferase assay (Fig. S6 and Table S3) is more reliable as the experiment was performed under dilute conditions where homodimerization is minimized. nd: not determined

^2^Interaction affinity was too low to be determined with reasonable accuracy.

**Table S2.** Fitted rate constants for heterodimerization reactions performed at 1 nM *vs.* 10 nM in lysate. Errors indicate standard deviations.

| **Design** | ***k*_obs_ (s^-1^)** |
| --- | --- |
| DHD37*^,1^ | 7 ± 3・10^-6^ |
| LHD29 | 3 ± 1・10^-4^ |
| LHD29* | 5.5 ± 2・10^-5^ |
| LHD274 | 1.40 ± 0.01・10^-3^ |
| LHD206 | 1.0 ± 0.5・10^-2^ |
| LHD202 | 1.8 ± 0.5・10^-2^ |
| LHD101-A53-B4 | 2.6・10^-2^ |
| LHD101 | 4.0 ± 0.1・10^-2^ |
| LHD101* | 4.2 ± 0.4・10^-2^ |

^1^[(Chen et al. 2019)](https://paperpile.com/c/raxPsK/R8rq). * Experiments performed with purified proteins, and reactions monitored by taking manual time-points as described in Materials and Methods and Supplementary Materials and Methods .

**Table S3.** Fitted equilibrium dissociation constants for binding curves collected in lysate. Errors indicate standard deviations.

| **Design** | ***K*_d_ (M)** |
| --- | --- |
| LHD101 | 2 ± 1・10^-8^ |
| LHD206 | 1.1 ± 0.4・10^-8^ |
| LHD101-A21-B82 | 1.1・10^-8^ |
| LHD29 | 6 ± 4・10^-8^ |
| LHD101-A53-B4 | 4 ± 1・10^-9^ |

**Table S4.** Crystallographic data collection and refinement.

|  | **LHD29 (PDB:6WMK)** | **LHD29A53/B53**  **(PDB: 7MWQ)** | **LHD101A53/B4**  **(PDB: 7MWR)** |
| --- | --- | --- | --- |
| **Data Collection** |  |  |  |
| Space group | *P 2_1_* | *P1* | *P 2_1_2_1_ 2_1_* |
| Cell dimensions |  |  |  |
| *a*, *b*, *c* (Å) | 56.07, 38.17, 60.37 | 61.31, 73.45, 4.14 | 45.40, 99.77, 122.09 |
| *α*, *β*, *γ* (°) | 90, 98.26, 90 | 108.39, 106.70, 110.15 | 90.0, 90.0, 90.0 |
| Resolution (Å) | 38.03 – 2.20 (2.42 - 2.20) | 51.56 - 2.56 (2.65 - 2.56) | 42.56 - 2.2 (2.27 - 2.20) |
| *R_merge_* (%) | 7 (56.9) | 8.3 (82.8) | 3.1 (49.2) |
| *R*_pim_ (%) | 4.6 (36.5) | 6.6 (69.5) | 3.1 (49.2) |
| *I/σ*(*I*) | 6.3 (1.4) | 4.7 (1.07) | 15.9 (1.6) |
| *CC* _1/2_ | 0.995 (0.705) | 0.991(0.651) | 0.999 (0.757) |
| Completeness (%) | 94.2 (99.2) | 97.9 (93.4) | 99.8 (99.0) |
| Redundancy | 3.3 (3.3) | 2.3 (2.4) | 2.0 (2.0) |
| **Refinement** |  |  |  |
| Resolution (Å) | 38.03 – 2.20 (2.42 - 2.20) | 51.56 - 2.56 (2.65 - 2.56) | 42.56 - 2.2 (2.27 - 2.20) |
| No. reflections | 12330 | 32540 | 28939 |
| *R*_work_ / *R*_free_ (%) | 25.3 / 28.3 (29.9 / 37.1) | 23.2 / 26.9 (36.9 / 41.9) | 21.1 /25.2 (40.6 /40.1) |
| No. atoms | 2154 | 6384 | 3514 |
| Protein | 2105 | 6370 | 11544 |
| Ligand | n/a | n/a | 7 |
| Water | 49 | 14 | 82 |
| Ramachandran  Favored/allowed  Outlier (%) | 96.80/3.20 | 98.64 / 1.11  0.25 | 97.77 /2.23  0.00 |
| R.m.s. deviations |  |  |  |
| Bond lengths (Å) | 0.002 | 0.002 | 0.002 |
| Bond angles (°) | 0.394 | 0.40 | 0.41 |
| *B*_factors_ (Å^2^) |  |  |  |
| Protein | 55.00 | 75.64 | 52.36 |
| Ligand | n/a | n/a | 78.04 |
| Water | 42.13 | 53.18 | 53.31 |

Data were collected from one crystal per condition. ^a^ Values given in parentheses refer to reflections in the outer resolution shell. For calculation of *R*_free_, 5% of all reflections were omitted from refinement.

**Table S5.** Comparative metrics designed models vs crystal structures

| **Design** | **RMSD** | **TMscore** | **lDDT** |
| --- | --- | --- | --- |
| LHD29 | 0.71 | 0.94 | 0.82 |
| LHD29A53/B53 | 3.4 | 0.85 | 0.80 |
| LHD101A53/B4 | 1.92 | 0.95 | 0.82 |

The root-mean-square deviation (RMSD) was calculated for C-alpha atoms. An RMSD score of 0 would indicate identical backbone C-alpha positions between crystal structure and design model. TMscore is the template modeling score according to Zhang and Skolnick [(*53*)](https://paperpile.com/c/He2YtO/4sYp+elsY), and lDDT (Local Distance Difference Test) is a superposition-free score that evaluates local distance differences of all atoms in a model [(*54*)](https://paperpile.com/c/He2YtO/4sYp+elsY). TMscore and lDDT can have values between 0 and 1, where 1 would indicate a perfect match.

**Data S1. DataS1_components_and_assemblies.xlsx**

Spreadsheet with sequences and parameters for proteins and assemblies shown in this work. Tab1: LHD components. Sequences and parameters for all heterodimers, fusions, connectors, and hubs presented in this work. Tab2: Mammalian cell constructs. Information on constructs used in transient transfection assays. Tab3: Luciferase constructs. Sequences and parameters of the proteins used in the split luciferase assay. Tab4: experimentally_validated_assemblies. List that specifies components of all linear assemblies shown in this work. Tab5: all_theoretical_assemblies. List of potential linear oligomers that could be assembled from the components shown in this work.

**Data S2. DataS2_pdbs_and_scripts.tar.gz**

Archive containing pdbs of components and assemblies shown in this work, as well as computational design scripts used to generate the heterodimers presented in this work.
